# Bottom-up assembly of graphene-decorated d-MXene nanoarchitecture for enhanced antibacterial activity against *Escherichia coli* and *Bacillus cereus*

**DOI:** 10.3762/bjnano.17.100

**Published:** 2026-10-22

**Authors:** Avdhesh Kumar, Ankit Singh, Ishani Bharti, Chandra Bhushan, Ram Naraian, Manish Pratap Singh

**Affiliations:** 1 Ionic Liquid and Nano Materials Laboratory, Department of Physics, Faculty of Engineering and Technology, V. B. S. Purvanchal University, Jaunpur, India; 2 Department of Biotechnology, V. B. S. Purvanchal University, Jaunpur, India

**Keywords:** *Bacillus cereus*, enhanced antibacterial activity, *Escherichia coli*, nanoarchitecture, non-covalent self-assembly

## Abstract

A bottom-up ultrasonication-assisted strategy was employed for the assembly of graphene-decorated d-MXene nanoarchitectures, and their antibacterial activity against *Escherichia coli* and *Bacillus cereus* was systematically investigated. The formation of the nanoarchitecture was confirmed through complementary morphological, structural, spectroscopic, and surface charge analyses. Scanning electron microscopy, elemental mapping, and high-resolution transmission electron microscopy observations revealed the uniform decoration of graphene nanosheets over layered d-MXene sheets, resulting in a structurally open architecture with suppressed nanosheet restacking. Powder X-ray diffraction (PXRD) analysis exhibited peak broadening, intensity variation, and modification of the low-angle (002) reflection, while comparative PXRD analysis with a composition-matched physical mixture further demonstrated disruption of the d-MXene stacking order following ultrasonication-assisted assembly. Raman spectroscopy exhibited the coexistence of graphene D (1339 cm^−1^) and G (1581 cm^−1^) bands together with the characteristic d-MXene vibrational mode at 153 cm^−1^ confirming preservation of defect-associated graphene domains within the assembled nanoarchitecture. Comparative Raman analysis further revealed systematic shifts in the characteristic graphene and d-MXene bands and a decrease in the *I*_D_/*I*_G_ ratio from 1.83 for the physical mixture to 1.27 for the nanoarchitecture, supporting intimate interfacial organization. X-ray photoelectron spectroscopy analysis revealed the coexistence of sp^2^ C=C (284.1 eV), sp^3^ C–C (284.7 eV), Ti–C (282.5 eV), and Ti–O (530.5 eV) bonding states, suggesting effective interfacial coupling and defect enriched surface regions after nanoarchitecture formation. Zeta potential analysis demonstrated enhanced colloidal stability of the graphene-decorated d-MXene nanoarchitecture with a highly negative surface charge value of −84.04 mV compared to graphene nanosheets (−54.7 mV) and d-MXene (−44.98 mV), indicating enhanced electrostatic stabilization and improved dispersion stability after nanoarchitecture formation. Dynamic light scattering analysis additionally revealed a reduction in average hydrodynamic size from 1658 nm for pristine d-MXene to 1211 nm after graphene incorporation, confirming suppression of nanosheets agglomeration within the assembled nanoarchitecture. The antibacterial activity was evaluated using colony-forming unit (CFU) and OD_600_ growth assays. The graphene-decorated d-MXene nanoarchitectures exhibited concentration-dependent antibacterial activity, reducing bacterial viability from 4.00 × 10^5^/4.20 × 10^5^ to 1.80 × 10^4^/2.10 × 10^4^ CFU/mL after 24 h at 500 µg/mL. Similarly, bacterial growth was significantly suppressed, with OD_600_ decreasing from 2.54/2.80 for untreated *E. coli*/*B. cereus* to 0.54/0.59 after treatment. Compared to a recent report (Ag/Ti_3_CNT*_x_*), the present system shows ≈39% relative reduction in *E. coli* viability without requiring silver doping. The enhanced activity is attributed to the cooperative contribution of improved nanosheet accessibility, membrane contact probability, exposed active interfacial domains, and a possible additional ROS-mediated pathway.

## Introduction

Rising rates of antibiotic-resistant bacterial infections have driven sustained interest in nanomaterial-based antibacterial strategies as alternatives or complements to conventional antibiotics, owing to their multi-target modes of action, which reduce the likelihood of resistance development compared with single-target antibiotic mechanisms [1]. As an initial step toward this goal, standard, non-resistant laboratory strains are typically used to establish baseline antibacterial efficacy and mechanism before extension to clinically resistant isolates. *Escherichia coli* (*E. coli*, Gram-negative) and *Bacillus cereus* (*B. cereus*, Gram-positive), used here as such model organisms, are established for antibacterial evaluation, differing fundamentally in cell-envelope architecture: *E. coli* possesses a thin peptidoglycan layer sandwiched between an outer and inner membrane, whereas *B. cereus* possesses a thicker peptidoglycan wall without an outer membrane. This structural distinction makes simultaneous evaluation against both strains essential for assessing the generality of an antibacterial mechanism [2–3]. Two-dimensional (2D) nanomaterials [4] have emerged as particularly effective antibacterial agents owing to their high surface-to-volume ratio, ultrathin morphology, and strong physical and chemical interaction capability with bacterial membranes [5–8]. Their antibacterial efficiency, however, depends not only on intrinsic composition but on nanosheet accessibility, aggregation state, exposed edge density, and colloidal dispersion behaviour [9–13], reflecting a broader shift in the field from identifying intrinsically antibacterial materials toward understanding how nanoscale structural organization governs the exposure of membrane-contact-active interfaces and bacterial interaction efficiency [14].

Graphene is a well-established antibacterial material owing to its atomically sharp nanosheet edges, defect-rich basal planes, high electrical conductivity, and excellent mechanical stability [15–17]. Its antibacterial action arises through multiple concurrent mechanisms, including direct membrane deformation and piercing by exposed edges, extraction of membrane phospholipids onto the graphene surface, and generation of oxidative stress at points of nanosheet–membrane contact [18]. MXenes, particularly Ti_3_C_2_T*_x_*-based systems, constitute a more recently developed class of 2D transition-metal carbides that exhibit substantial antibacterial activity owing to their metallic conductivity, hydrophilic surfaces, and abundant terminal functional groups (–O, –OH, –F), which promote electrostatic interaction with negatively charged bacterial membrane components [19–20]. A key advantage of MXenes over other 2D antibacterial materials is the tunability of their surface termination chemistry and interlayer spacing through the etching and delamination conditions used during synthesis, offering an additional design axis for antibacterial optimization beyond composition alone [21–22].

Because graphene and MXenes independently exhibit antibacterial activity, graphene–MXene hybrid systems have already been explored, primarily for multifunctional applications spanning electrochemical energy storage, electromagnetic interference shielding, biosensing, photothermal therapy, and antibacterial coatings [23–25]. However, in most previously reported graphene–MXene antibacterial systems, enhanced activity is interpreted as a simple additive consequence of combining two independently antibacterial materials, without systematic investigation of how nanosheet organization, colloidal stability, and interfacial exposure collectively govern the resulting antibacterial behaviour [26]. This additive framing overlooks a critical limitation common to both constituent materials: Graphene and MXene nanosheets are highly prone to aggregation and restacking during dispersion and processing. Graphene nanosheets experience strong π–π stacking and van der Waals attraction between adjacent basal planes, leading to irreversible agglomeration and loss of accessible, defect-rich active edges. MXene nanosheets similarly undergo interlayer restacking driven by van der Waals forces and hydrogen bonding between surface terminations, substantially reducing accessible surface area and suppressing membrane-contact probability even at high loading concentrations. Such aggregation-induced structural collapse can significantly diminish bacterial interaction efficiency regardless of the intrinsic antibacterial activity of the individual components, meaning that simply combining graphene with MXene does not guarantee enhanced antibacterial performance unless the resulting architecture actively preserves accessible interfacial regions under dispersed, aqueous conditions. Recent reviews have accordingly highlighted suppression of nanosheet restacking and enhancement of exposed interfacial density as key parameters governing antibacterial efficiency in MXene-based systems, implicating the nature of non-covalent self-assembly, rather than composition alone, as a determining factor in antibacterial performance.

Despite this recognition, a clear gap remains in the graphene–MXene antibacterial literature. Existing studies rarely benchmark hybrid antibacterial performance systematically against the individual graphene and MXene constituents under identical experimental conditions, and comparatively little attention has been paid to how non-covalent self-assembly modifies hydrodynamic aggregation, electrostatic surface characteristics, and interfacial accessibility in graphene-decorated delaminated MXene systems specifically [27]. More fundamentally, because graphene–MXene composites are typically graphene-rich by design, it remains unresolved in the existing literature whether reported antibacterial enhancements reflect genuine interfacial assembly effects or simply the dominant graphene fraction of the hybrid [28–29]. To our knowledge, no prior study has directly addressed this ambiguity using a composition-matched physical mixture control, without which an observed antibacterial enhancement cannot be confidently attributed to interfacial organization rather than composition ratio.

In this work, graphene-decorated d-MXene nanoarchitectures were assembled via ultrasonication-assisted non-covalent self-assembly, and their antibacterial activity against *E. coli* and *B. cereus* was evaluated using a multimodal experimental strategy including structural and compositional confirmation by scanning electron microscopy (SEM), high-resolution transmission electron microscopy (HRTEM), selected area electron diffraction (SAED), powder X-ray diffraction (PXRD), Raman spectroscopy, and X-ray photoelectron spectroscopy (XPS), colloidal characterization by zeta potential measurements and dynamic light scattering, antibacterial evaluation by counting colony-forming units (CFUs), measuring OD_600_ growth, and viability assays, and mechanistic decomposition of membrane-associated versus reactive oxygen species (ROS)-mediated antibacterial contributions via a nitro blue tetrazolium (NBT)-based superoxide assay. Critically, PXRD and Raman analyses were additionally performed on a composition-matched physical mixture control to directly test whether the observed structural and spectroscopic differences reflect genuine interfacial coupling rather than the graphene-rich composition ratio alone. The novelty of this work therefore lies not in graphene–MXene hybridization itself, but in demonstrating, through composition-matched controls and mechanistic pathway decomposition, that the resulting antibacterial enhancement is governed predominantly by interfacial organization and dispersion-state control rather than by additive material combination or composition ratio. Beyond the specific system studied here, this work establishes a structure–property framework and a broadly applicable control-based methodology for distinguishing genuine interfacial coupling from compositional artefacts in other 2D hybrid antibacterial systems, with relevance to the design of antibacterial coatings, wound dressings, water purification membranes, and food packaging materials.

## Results and Discussion

### Morphological analysis

**Figure 1:**
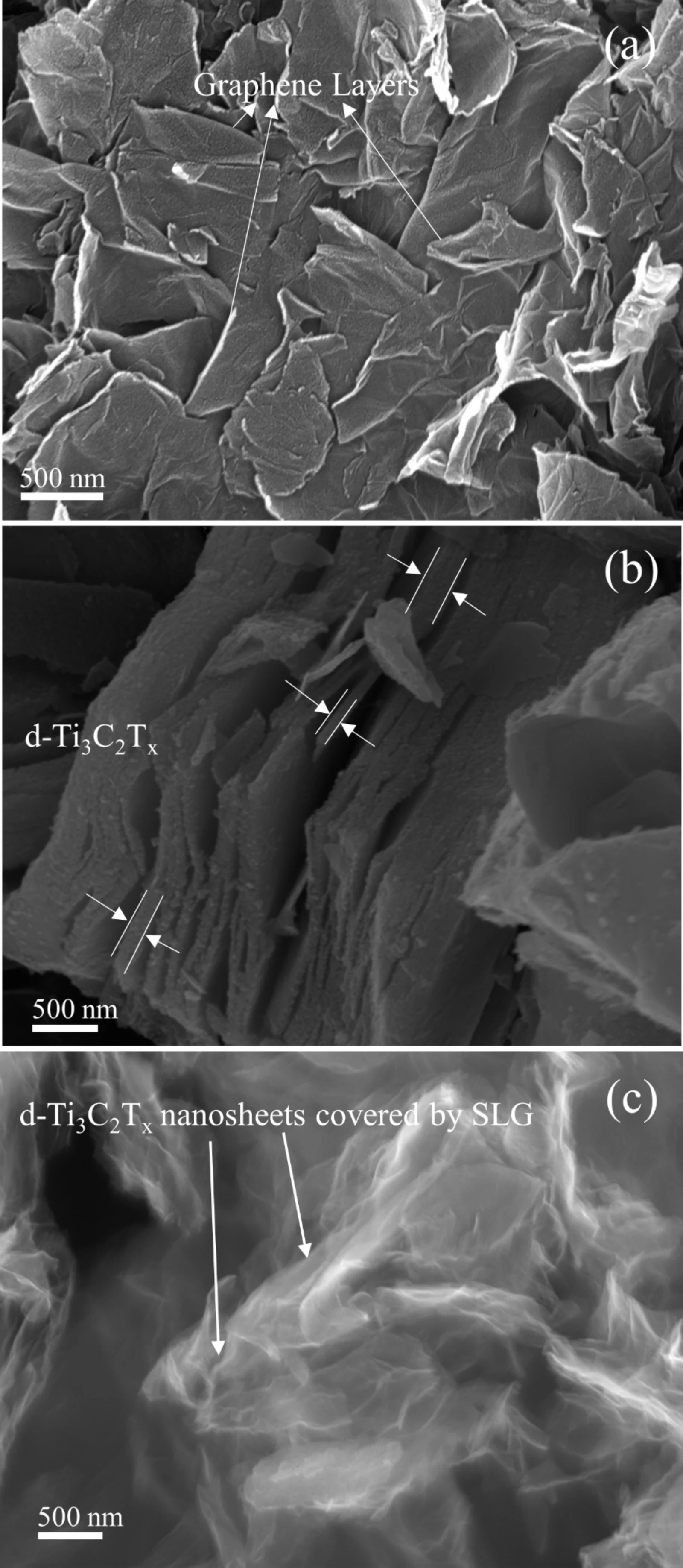
FE-SEM micrographs of (a) graphene nanosheets, (b) d-MXene, and (c) the graphene-decorated d-MXene nanoarchitectures.

Figure 1 displays the FE-SEM morphologies of graphene nanosheets, d-MXene, and graphene-decorated d-MXene nanoarchitectures. In Figure 1a, graphene nanosheets exhibit the characteristic thin, wrinkled, and crumpled sheet-like morphology, appearing as randomly distributed flakes with sharp edges and overlapping regions. Such features are typical for graphene nanosheets due to their ultrathin nature and high flexibility. The observed thickness (10–15 nm) corresponds to stacked or folded graphene nanosheets regions, confirming the graphene in a nanosheet form with high exposed surface area. In Figure 1b, the d-MXene exhibits a distinct multilayer accordion-like morphology with expanded interlayer spacing, confirming successful etching and delamination of the Ti_3_AlC_2_ MAX phase. The stacked lamellar structure with open galleries is a well-established signature of Ti_3_C_2_T*_x_* MXene after Al removal and exfoliation. The average thickness of d-MXene stacks is observed to be ≈65 nm, indicating multilayer sheets with accessible interlayer voids. Most importantly, the FE-SEM image of the graphene-decorated d-MXene nanoarchitectures (Figure 1c) demonstrates a morphology that is qualitatively different from both pristine graphene nanosheets and pristine d-MXene, confirming the creation of an integrated architecture. In this image, the wrinkled and semitransparent graphene sheets are clearly seen wrapping and covering the d-MXene nanosheet domains, forming continuous interfacial contact over large regions. Unlike a random mixture, where graphene nanosheets flakes and d-MXene layers would appear as separated, independently stacked particles-the graphene-decorated d-MXene nanoarchitectures shows co-localization and interpenetration of the two phases; graphene nanosheets are observed in close contact with the d-MXene surface and interlayer regions, consistent with the formation of a non-covalently assembled nanoarchitecture. This surface coverage suggests that graphene and d-MXene are assembled through close interfacial contact rather than existing as completely separated nanosheets. Under the present synthesis conditions, this assembly is expected to arise predominantly from non-covalent interactions between the two components. Additionally, the graphene-decorated d-MXene nanoarchitectures exhibits suppressed MXene restacking and increased interlayer openness, evidenced by the less compact, more expanded hierarchical arrangement compared to pristine d-MXene.

This morphological transformation strongly supports the role of graphene nanosheets as a non-covalent interfacial spacer, preventing dense lamellar collapse of MXene layers through physical interposition between and over the d-MXene sheets. The resulting architecture, therefore, represents a genuine 2D/2D stacked graphene-decorated d-MXene nanoarchitectures, where graphene sheets are distributed between and over d-MXene layers and stabilize an expanded, interconnected network. Thus, FE-SEM analysis provides three key proofs for graphene-decorated d-MXene nanoarchitectures formation: (i) direct visualization of graphene nanosheets sheets coating and interconnecting d-MXene nanosheets, (ii) a distinct hierarchical morphology not present in either pristine component, and (iii) clear reduction in MXene restacking with enhanced structural openness, all of which are consistent with the successful formation of the graphene-decorated d-MXene nanoarchitecture. This intimate interfacial integration is expected to enhance antibacterial activity by maximizing exposed sharp edges, increasing accessible surface-active sites, and promoting interfacial interactions beneficial for oxidative stress pathways.

### Elemental composition and elemental mapping analysis

**Figure 2:**
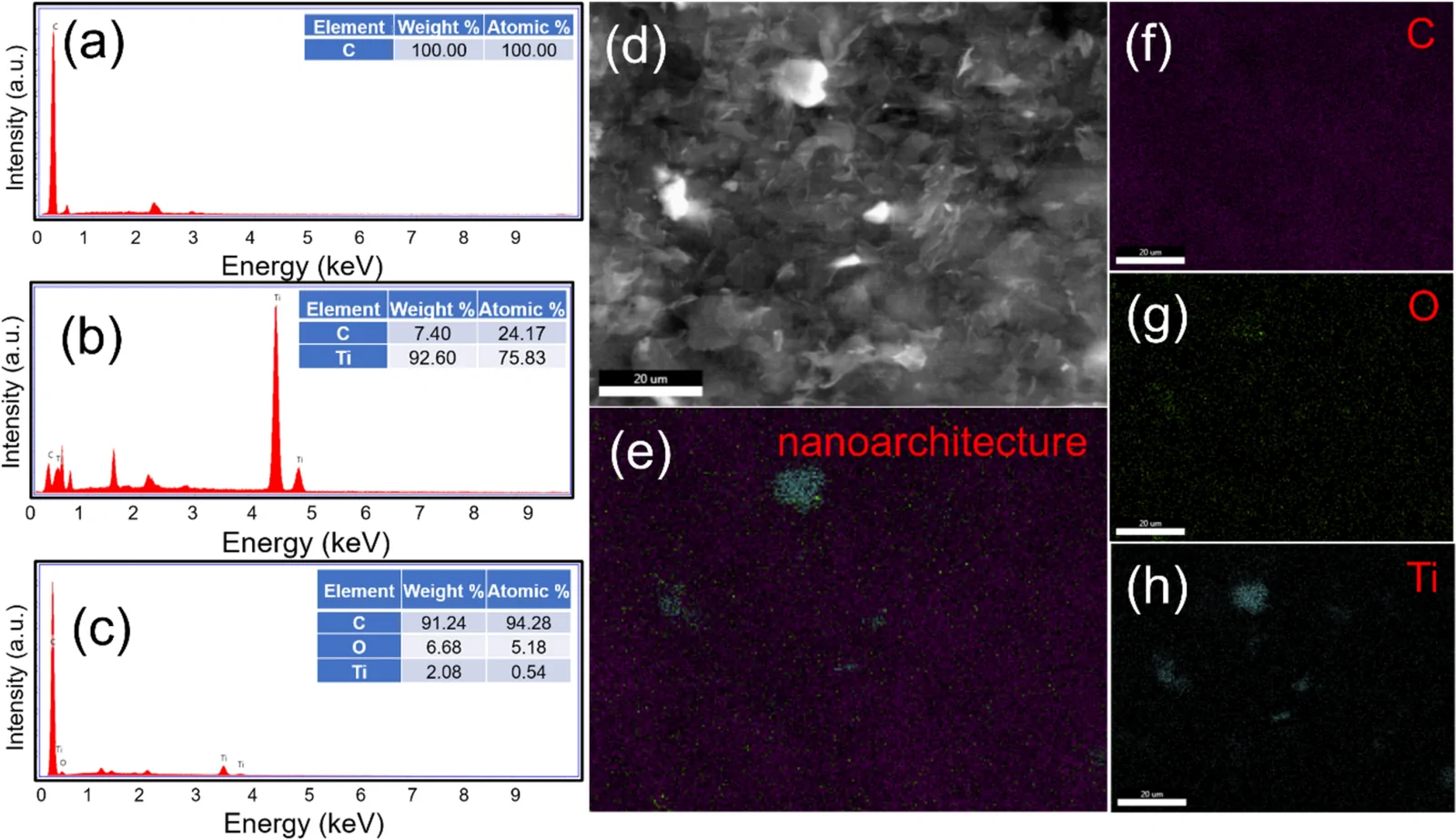
EDS spectra of (a) graphene, (b) d-MXene, and (c) graphene-decorated d-MXene nanoarchitecture. (d) SEM image and corresponding EDS elemental mapping of the graphene-decorated d-MXene nanoarchitecture showing (e) overlay, (f) C, (g) O, and (h) Ti elemental distributions.

To further verify the elemental composition and spatial distribution of the constituent elements within the graphene-decorated d-MXene nanoarchitecture, energy-dispersive X-ray spectroscopy (EDS) and elemental mapping analyses were performed, as shown in Figure 2. The EDS spectrum of graphene nanosheets (Figure 2a) exhibits a single intense carbon peak, confirming the high purity of the graphene nanosheets without detectable metallic impurities. Quantitative analysis indicates that the sample consists of 100 wt % carbon, consistent with the graphitic nature of the material. The EDS spectrum of d-MXene (Figure 2b) shows prominent Ti and C peaks, confirming the successful formation of d-MXene. Quantitative analysis gives 92.60 wt % Ti and 7.40 wt % C, while no Al signal is detected, indicating the effective removal of the Al atomic layers during the HF etching process. The absence of Al further confirms the successful conversion of the MAX phase into d-MXene. The EDS spectrum of the graphene-decorated d-MXene nanoarchitecture (Figure 2c) simultaneously exhibits C, Ti, and O peaks, confirming the coexistence of graphene and d-MXene within the assembled nanoarchitecture. Quantitative analysis reveals elemental contents of 91.24 wt % C, 2.08 wt % Ti, and 6.68 wt % O, corresponding to atomic percentages of 94.28 atomic % (atom %) C, 0.54 atom % Ti, and 5.18 atom % O, respectively. The predominance of carbon is expected because the nanoarchitecture was intentionally synthesized using a graphene-rich composition, whereas the Ti signal confirms the incorporation of the d-MXene component. The oxygen signal originates from the environmental oxidation and oxygen-containing surface terminations (–O and –OH). To further investigate the spatial distribution of the constituent elements, FE-SEM-EDS elemental mapping was carried out for the graphene-decorated d-MXene nanoarchitecture (Figure 2d–h). The FE-SEM image (Figure 2d) reveals the sheet-like morphology of the assembled nanoarchitecture, while the corresponding overlay elemental map (Figure 2e) demonstrates the simultaneous distribution of carbon, titanium, and oxygen throughout the entire observed region. The individual elemental maps of C (Figure 2f), O (Figure 2g), and Ti (Figure 2h) reveal that all three elements are uniformly distributed without noticeable elemental segregation.

The homogeneous spatial distribution of Ti within the continuous carbon-rich matrix indicates that the d-MXene nanosheets are uniformly dispersed throughout the graphene framework rather than existing as localized aggregates. This observation is consistent with the proposed graphene-decorated d-MXene nanoarchitecture and supports the formation of nanoarchitecture.

### HRTEM and SAED analysis

**Figure 3:**
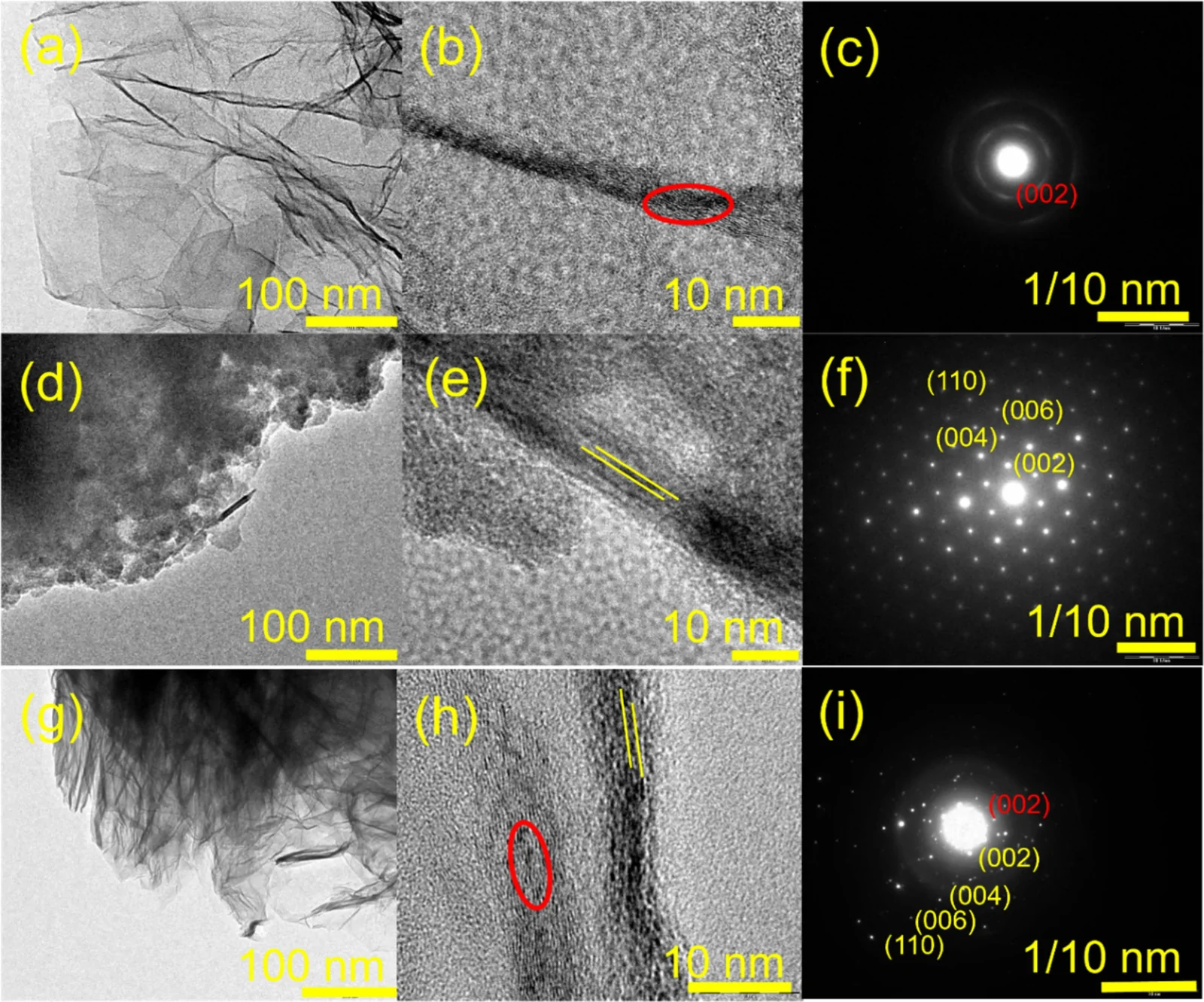
TEM, HRTEM, and SAED characterization of (a–c) graphene, (d–f) d-MXene, and (g–i) graphene-decorated d-MXene nanoarchitecture.

The microstructure of the synthesized materials was further examined to provide direct structural evidence for the graphene-decorated d-MXene nanoarchitecture, HRTEM together with SAED analyses were performed, as shown in Figure 3. The TEM image of graphene nanosheets (Figure 3a) reveals ultrathin, highly transparent, wrinkled two-dimensional sheets with numerous folded edges, characteristic of few-layer graphene. The high transparency indicates the ultrathin nature of the nanosheets, while the wrinkles originate from the intrinsic flexibility of graphene. The corresponding HRTEM image (Figure 3b) further confirms the well-ordered graphitic structure through clearly resolved lattice fringes (highlighted, red oval) extending along the sheet. The SAED pattern (Figure 3c) exhibits a diffuse diffraction ring indexed to the graphitic (002) plane, confirming the crystalline nature of the graphene nanosheets, consistent with the turbostratic stacking typical of few-layer graphene.

The TEM image of d-MXene (Figure 3d) displays thin layered nanosheets with a transparent morphology, confirming successful exfoliation of the d-MXene sheets after chemical etching and delamination. Unlike the compact multilayer morphology of the MAX phase, the sheets are loosely stacked with enlarged interlayer regions. The corresponding HRTEM image (Figure 3e) reveals well-resolved lattice fringes (highlighted, yellow lines) extending continuously along the d-MXene layers, indicating that the crystal structure is preserved after delamination. The SAED pattern (Figure 3f) consists of sharp diffraction spots indexed to the (002), (004), (006), and (110) planes of d-MXene, confirming its high crystallinity.

A distinctly different morphology is observed for the graphene-decorated d-MXene nanoarchitecture (Figure 3g). Thin graphene nanosheets are observed in intimate contact with the d-MXene layers, producing a continuous interconnected 2D structure. The graphene sheets are uniformly distributed over the exposed d-MXene surface and are also observed between d-MXene layers, giving rise to a more open layered architecture, consistent with suppression of d-MXene restacking following graphene incorporation. The corresponding HRTEM image (Figure 3h) provides direct visualization of the interface between graphene and d-MXene. Notably, this image simultaneously captures the graphene-characteristic lattice fringe (red oval, consistent with Figure 3b) and the MXene-characteristic interlayer spacing (yellow lines, consistent with Figure 3e) within a single field of view, directly demonstrating that both crystal lattices coexist in intimate nanoscale contact while each retains its individual structural identity. Such close interfacial contact confirms the formation of the nanoarchitecture through non-covalent interactions during ultrasonication-assisted self-assembly.

The SAED pattern of the nanoarchitecture (Figure 3i) simultaneously exhibits two distinct (002)-related features, namely, a diffuse ring characteristic of graphene (consistent with Figure 3c) and a sharp spot characteristic of d-MXene, appearing alongside the additional (004), (006), and (110) MXene reflections (consistent with Figure 3f). The clear separation of these two (002) signals, rather than their merging into a single shared reflection, confirms that the crystalline structures of graphene and d-MXene remain structurally distinct and are preserved independently after assembly, consistent with a non-covalently coupled interfacial architecture rather than a new epitaxial phase.

### Structural analysis

**Figure 4:**
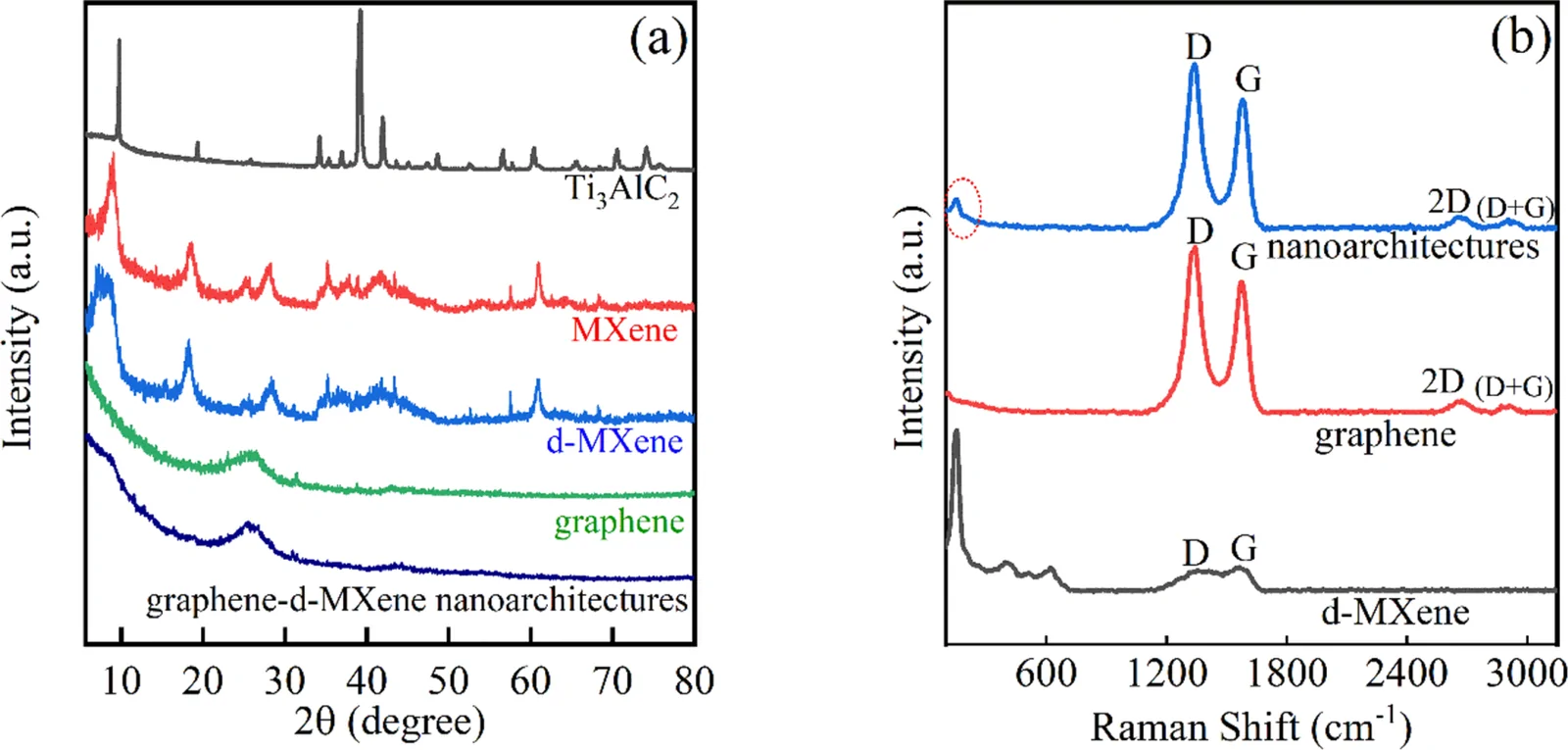
(a) PXRD patterns of the Ti_3_AlC_2_ MAX phase, etched Ti_3_C_2_T*_x_*, delaminated Ti_3_C_2_T*_x_* (d-MXene), graphene nanosheets, and graphene-decorated d-MXene nanoarchitectures. (b) Raman spectra of graphene nanosheets, d-MXene, and graphene-decorated d-MXene nanoarchitectures.

Figure 4a presents the PXRD patterns of the Ti_3_AlC_2_ MAX phase, etched Ti_3_C_2_T*_x_*, delaminated Ti_3_C_2_T*_x_* (d-MXene), graphene nanosheets, and the graphene-decorated d-MXene nanoarchitectures recorded in the 2θ range of 5–80° at room temperature. PXRD was employed to confirm (i) selective etching of the Al layer from Ti_3_AlC_2_, (ii) successful conversion into d-MXene and subsequent delamination, and (iii) non-covalent self-assembly of graphene nanosheets with d-MXene to form a graphene-decorated d-MXene nanoarchitectures. The Ti_3_AlC_2_ MAX phase exhibits sharp and well-defined reflections at 9.8°, 19.4°, 34.2°, 36.9°, 39.2°, 41.9°, 48.6°, 52.4°, 56.6°, 60.4°, and 65.5°, corresponding to the lattice planes (002), (004), (101), (102), (104), (105), (106), (107), (108), (110), and (1011), respectively. These peaks agree well with the standard JCPDS data for Ti_3_AlC_2_ (JCPDS No. 05-0875), confirming phase purity of the starting MAX material. After HF etching, the diffraction pattern of Ti_3_C_2_T*_x_* shows a pronounced change compared to Ti_3_AlC_2_, demonstrating successful removal of Al layers. The characteristic low-angle reflection shifts and appears at ≈8.7°, while additional peaks are observed at 18.6°, 27.9°, 35.2°, 41.7°, 57.6°, and 60.9°, consistent with the formation of the layered Ti_3_C_2_T*_x_* structure. Importantly, the Al-associated MAX peak at 39.2° (104) significantly decreases or disappears after etching, indicating efficient elimination of Al from the parent MAX phase. The disappearance/weakening of other MAX-related reflections further confirms the conversion from Ti_3_AlC_2_ into MXene. Following DMSO-assisted delamination, the (002) peak of Ti_3_C_2_T*_x_* shifts further toward lower angles from 8.7° to 7.7°, which is a direct signature of interlayer expansion and successful delamination. This low-angle shift corresponds to an increased interlayer distance due to solvent intercalation and separation of stacked MXene sheets. In addition, slight shifts in reflections near 18.6° and 27.9°, along with broadening of the most intense peaks, further confirm a reduced stacking order and enhanced exfoliation of Ti_3_C_2_T*_x_* into d-MXene. The PXRD pattern of d-MXene matches well with the reference file (JCPDS No. 96-722-1325), validating the crystalline phase of delaminated MXene. The PXRD pattern of graphene nanosheets exhibit a broad characteristic peak at ≈26.2°, corresponding to the (002) plane of graphitic carbon, confirming the presence of layered graphene sheets. A weak additional peak at ≈42.1° is attributed to the (100) plane, further supporting the graphitic structure.

Most notably, the graphene-decorated d-MXene nanoarchitectures exhibits diffraction features originating from both graphene and d-MXene. The diffraction pattern is dominated by the graphitic (002) reflection at 26.2°, whereas the low-angle MXene (002) reflection appears comparatively weak because of the high graphene-to-d-MXene mass ratio employed during synthesis. Nevertheless, compared with pristine MXene, the d-MXene (002) reflection becomes broader and less intense, indicating reduced stacking order and structural modification of the layered d-MXene framework following graphene incorporation. These PXRD observations alone are not sufficient to unequivocally distinguish non-covalent self-assembly from a simple physical mixture.

The interlayer spacing (*d*) of graphene nanosheets, d-MXene, and the graphene-decorated d-MXene nanoarchitecture was calculated using Bragg’s law [8] (Equation 1):


[1] 2dsinθ=nλ,

where *n* is the order of reflection (*n* = 1), λ is the X-ray wavelength (Cu Kα, λ = 1.54 Å), *d* is the interlayer spacing, and θ is the Bragg diffraction angle. The (002) reflection of Ti_3_C_2_T*_x_* at 2θ = 8.7° corresponds to *d* = 1.016 nm, which increases to *d* = 1.147 nm after delamination (2θ = 7.7°), confirming a clear expansion of the interlayer distance due to intercalation and exfoliation. In contrast, graphene nanosheets display the characteristic (002) reflection at 2θ = 26.2° with *d* = 0.340 nm, consistent with graphitic carbon.

The lattice parameter (*c*) of the Ti_3_C_2_T*_x_* structure was subsequently determined from the (002) reflection according to Equation 2, assuming a hexagonal crystal structure:


[2] $$c=2d_{002},$$

where *c* is the lattice parameter along the *c* axis and *d*_002_ is the interlayer spacing calculated from Bragg’s law for the (002) reflection. The increase in d-spacing and broadening of the low-angle peak in the graphene-decorated d-MXene further supports successful nanoarchitectures assembly with reduced restacking and enhanced interfacial interaction. Furthermore, the crystallite size was estimated using the Scherrer equation [30] (Equation 3):


[3] $$D=\frac{K\lambda }{\beta \cos\theta },$$

where *D* is crystallite size, *K* is a constant, λ is the X-ray wavelength (1.54 Å), θ is the Bragg diffraction angle (in radians), and β is the full width at half maximum (FWHM) of the diffraction peak (in radians) [31]. The dislocation density (δ) of the crystal [31] was calculated using Equation 4:


[4] $$\delta =\frac{1}{D^{2}},$$

where δ is the dislocation density and *D* is the crystallite size obtained from the Scherrer equation (Equation 3) [32].

The process of etching and delamination shows defects in crystals. Furthermore, the crystallite sizes calculated from Equation 3 were 18.970 nm (graphene nanosheets), 71.822 nm (d-MXene), and 33.975 nm (graphene nanosheets/d-MXene), with corresponding dislocation densities of 2.78 × 10^−3^, 1.94 × 10^−4^, and 8.66 × 10^−4^ nm^−2^, respectively, indicating structural modification of the nanoarchitectures. PXRD-derived structural parameters of graphene nanosheets, Ti_3_C_2_T*_x_*, d-MXene, and graphene-decorated d-MXene nanoarchitectures are listed in Table 1.

**Table 1:** PXRD-derived structural parameters of graphene nanosheets, Ti_3_C_2_T*_x_*, d-MXene, and graphene-decorated d-MXene nanoarchitectures.

Material	2θ (°)	Crystallite size (*D*) (nm)	Interplanar spacing (*d*) (nm)	Lattice parameter (*c*) for the hexagonal cell (nm)	Dislocation density (δ) (nm^−2^)

graphene nanosheets	26.2°	18.970	0.340	0.680	2.78 × 10^−3^
Ti_3_C_2_T_x_	8.7°	71.822	1.016	2.032	1.93× 10^−4^
d-MXene	7.7°	28.760	1.147	2.294	1.94 × 10^−4^
nanoarchitectures	26.2°	33.975	0.340	0.680	8.66 × 10^−4^

Comparative PXRD measurements were performed on the ultrasonication-assisted nanoarchitecture and a physical mixture prepared using the same graphene and d-MXene composition (Figure S1, Supporting Information File 1), in order to further verify that the graphene-decorated d-MXene material is not merely a simple physical mixture. Both samples exhibit a broad reflection centred at 26.2°, corresponding to the characteristic graphitic (002) plane of graphene, consistent with the intentionally graphene-rich composition of the nanoarchitecture. As the characteristic d-MXene (002) reflection is typically reported near 2θ = 6–9°, it is not clearly resolved within the presently scanned 2θ range and is obscured by the dominant graphene signal. Distinct differences between the nanoarchitecture and the physical mixture are nonetheless observed in the 20–30° region, where the nanoarchitecture displays a sharper and more resolved graphitic (002) reflection relative to the physical mixture. This sharpening suggests improved graphitic stacking order arising from the interfacial assembly process, rather than reflecting a separate MXene contribution. A weak additional peak observed at ≈43° in the nanoarchitecture, assigned to the (100) plane of graphitic carbon, is attributed to minor oxidation occurring during the sonication process. In contrast, the physical mixture retains a comparatively broad and less resolved (002) feature, consistent with the individual components retaining their native, loosely associated stacking without genuine interfacial coupling.

### Defect and vibrational analysis

Figure 4b shows the Raman spectra of graphene nanosheets, d-MXene, and the graphene-decorated d-MXene nanoarchitectures, confirming the successful formation of each component and providing strong evidence for the successful assembly of the nanoarchitecture through the coexistence and evaluation of their characteristic vibrational bands. The Raman spectrum of graphene nanosheets exhibit two prominent bands at 1335 cm^−1^ (D band) and 1545 cm^−1^ (G band). The D band originates from the defect/disorder-induced breathing vibrations of sp^2^ carbon rings, whereas the G band corresponds to the in-plane stretching vibration of sp^2^-hybridized carbon atoms. The *I*_D_/*I*_G_ ratio of ≈1.27 indicates the presence of abundant structural defects and edge-rich domains, which are characteristic of ultrathin graphene nanosheets. In addition, weak second order Raman peaks look at 2766 cm^−1^ (2D) and 2909 cm^−1^ (D+G) further confirm the graphitic nature of graphene sheets [32].

For d-MXene the Raman spectrum exhibits characteristic vibrations associated with Ti_3_C_2_T*_x_* MXene, primarily located in the low-wavenumber region (<800 cm^−1^) and arising from Ti–C bands, Ti–T*_x_* surface terminations, and lattice vibrational modes. Notably, the disappearance of the characteristic band ≈270 cm^−1^, attributed to Al vibrations in Ti_3_C_2_T*_x_*, together with the emergence of Ti-related vibrational modes at 151, 387, and 617 cm^−1^, confirms the effective removal of Al layers and the successful formation of d-MXene [33]. The broad D (1344 cm^−1^) and G (1565 cm^−1^) bands are also observed, originating from carbon atoms within the d-MXene structure and residual carbon species. The measurement ratio of 0.95 indicates a moderate defect density, arising from delamination-induced structural distortions, including vacancies, grain boundaries, and amorphous carbon species [32,34].

Most importantly, the Raman spectrum of the graphene-decorated d-MXene nanoarchitectures simultaneously displays the characteristic Raman features of both graphene and d-MXene, including the Ti-related vibrational mode at 153 cm^−1^ together with the graphene D (≈1339 cm^−1^) and G (1581 cm^−1^) bands. The coexistence of the graphene D and G bands together with the low-frequency MXene vibrational mode indicates the presence of both constituents within the hybrid nanoarchitecture. Because the graphene-to-d-MXene mass ratio is intentionally high, the Raman spectrum is dominated by the graphene bands, while the MXene vibrational feature appears comparatively weak, which is consistent with the lower relative amount of d-MXene in the composite. Notably, the graphene-decorated d-MXene nanoarchitectures exhibit an *I*_D_/*I*_G_ ratio of ≈1.27, which is identical to that of pristine graphene. The observation indicates that the defect-rich and edge-abundant structure of graphene is largely preserved following assembly with d-MXene, suggesting that the fabrication process does not introduce significant additional structural defects. Instead, the slight spectral evolution is primarily attributed to possible interfacial interactions between graphene and d-MXene rather than defect generation. The preservation of the graphene D and G bands together with the d-MXene fingerprint region confirms the successful formation of an interfacially coupled nanoarchitecture rather than a simple physical mixture as both components retain their characteristic vibrational signatures while exhibiting subtle spectral evolution arising from electronic interactions at the heterointerface. The weak second-order 2D and (D+G) bands further support the graphitic nature of the graphene component and indicate that its two-dimensional framework remains structurally intact after assembly [32].

To confirm that this preserved graphitic ordering reflects genuine interfacial coupling rather than simply the graphene-rich composition ratio, the nanoarchitecture was further compared against a compositionally matched physical mixture (Figure S2, Supporting Information File 1). The nanoarchitecture exhibits a clearly enhanced band in the 1500–1700 cm^−1^ region relative to the physical mixture, along with subtle differences in the low-wavenumber and 2500–2900 cm^−1^ regions. More importantly, the *I*_D_/*I*_G_ ratio decreases from 1.83 for the physical mixture to 1.27 for the nanoarchitecture, a value essentially identical to that of pristine graphene. This indicates that the ultrasonication-assisted assembly does not introduce additional structural disorder into the graphene framework relative to its pristine state; instead, it suggests a more ordered graphitic structure arising from uniform interfacial organization and intimate contact between graphene and d-MXene nanosheets, while the elevated *I*_D_/*I*_G_ observed in the physical mixture reflects defects or disorder introduced by random sheet aggregation during simple mechanical blending, which the controlled assembly process avoids. Taken together with the PXRD results, these spectral differences are attributed to genuine interfacial coupling between graphene and d-MXene arising from the ultrasonication-assisted assembly process, rather than to compositional variation alone. The combination of preserved, defect-rich graphene and conductive d-MXene establishes an improved nanoarchitecture that contributes to the superior antibacterial activity observed for the graphene-decorated d-MXene nanoarchitecture.

### Elemental and electronic state analysis

**Figure 5:**
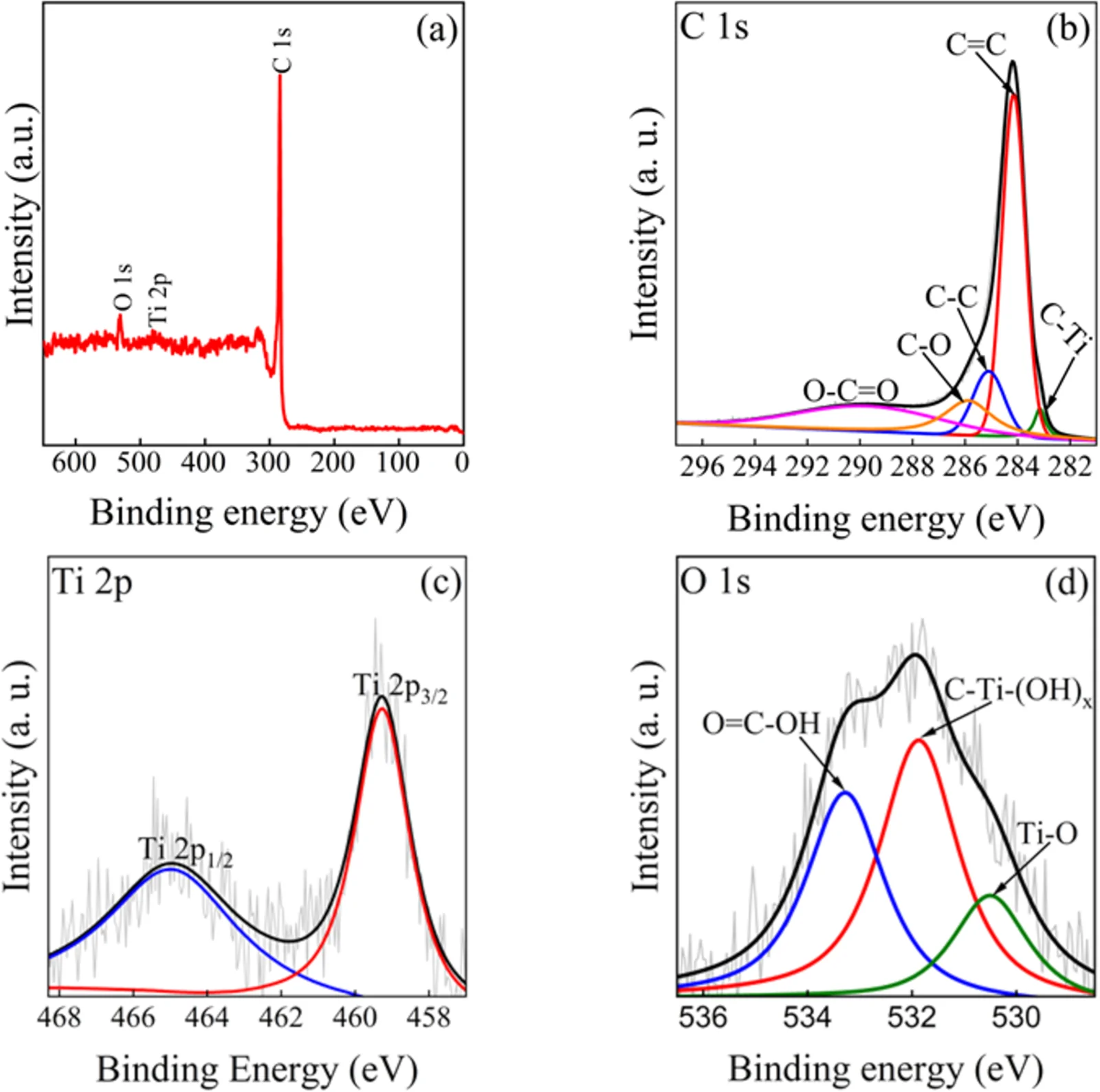
XPS analysis of the graphene-decorated d-MXene nanoarchitecture: (a) survey spectrum, (b) high-resolution C 1s spectrum, (c) high-resolution Ti 2p spectrum, and (d) high-resolution O 1s spectrum.

XPS was employed to investigate the surface elemental composition, chemical bonding environment, and oxidation states of the graphene-decorated d-MXene nanoarchitecture (Figure 5) [35]. The survey spectrum (Figure 5a) confirms the presence of C, Ti, and O as the principal surface elements. Quantitative analysis revealed atomic concentrations of C, Ti, and O of 96.26, 0.61, and 3.13 atom %, respectively. The predominance of carbon is consistent with the graphene-rich composition of the nanoarchitecture, whereas the Ti signal confirms the presence of the d-MXene framework, and the O signal comparatively higher than Ti reflects oxygen-containing surface terminations associated with both the d-MXene and residual graphene functional groups. The high-resolution C 1s spectrum (Figure 5b) was deconvoluted into five components located at 282.5, 284.13, 284.70, 286.10, and 290.20 eV, corresponding to C–Ti, C=C (sp^2^ carbon), C–C (sp^3^ carbon), C–O, and O–C=O, respectively. The dominant C=C peak confirms the graphitic framework of the graphene nanosheets, while the C–O and O–C=O components arise from residual oxygen-containing functional groups and surface terminations associated with the graphene-decorated d-MXene nanoarchitecture.

The C–Ti component, essentially unchanged in binding energy relative to the physical mixture control (282.4 eV; Supporting Information File 1, Figure S3b), is attributed to the native Ti–C carbide framework intrinsic to d-MXene, confirming that the MXene phase is retained within the nanoarchitecture rather than indicating formation of a new interfacial bond. This Ti–C component, falling within the literature-reported range for carbidic carbon in Ti_3_C_2_T*_x_* (typically 281.5–282.8 eV), constitutes the primary direct XPS-based evidence for retention of the Ti–C carbide framework in this study (see Ti 2p discussion below).

The high-resolution Ti 2p spectrum (Figure 5c) exhibits a spin–orbit doublet centered at 459.20 eV (Ti 2p_3/2_) and 465.03 eV (Ti 2p_1/2_). Based on comparison with literature binding energies for Ti_3_C_2_T*_x_* MXene, where the native Ti–C carbide component of Ti 2p_3/2_ is consistently reported near 454.7–455.3 eV, the observed doublet at 459.20 eV is instead consistent with an oxidized titanium environment (Ti–O/TiO_2_-like), rather than direct evidence of the underlying Ti–C carbide framework. This assignment is corroborated by the O 1s spectrum (Figure 5d), where a corresponding Ti–O component is resolved at 530.5 eV. Given the low overall Ti content detected by survey XPS (0.61 atom %) and the surface-sensitive nature of XPS (probing depth of a few nanometres), the Ti 2p signal is likely dominated by oxygen-terminated and/or partially oxidized Ti sites at the outermost surface of the d-MXene sheets, consistent with the native oxygen-containing surface terminations (–O, –OH) of Ti_3_C_2_T*_x_* and its known susceptibility to surface oxidation upon ambient air exposure, rather than reflecting the absence of the carbide core itself. Direct evidence for retention of the Ti–C carbide framework is instead provided by the C 1s spectrum (Figure 5b), where the C–Ti component at 282.5 eV falls within the literature-reported range for carbidic carbon in Ti_3_C_2_T*_x_*, and by the independent structural characterization of pristine d-MXene reported elsewhere in this study (PXRD, Figure 4a; Raman, Figure 4b; HRTEM/SAED, Figure 3d–f; EDS, Figure 2b), which collectively confirm successful conversion from the Ti_3_AlC_2_ MAX phase and retention of the layered carbide structure. We note that a lower-binding-energy Ti-C component (≈455 eV) was not resolved in the present Ti 2p spectrum, and we have revised our discussion accordingly to avoid over-attributing the observed Ti 2p doublet to the carbide framework.

The deconvoluted O 1s spectrum (Figure 5d) consists of three components centered at 530.5, 531.8, and 533.3 eV, assigned to Ti–O, C–Ti–(OH)*_x_*, and O=C–OH, respectively. The Ti–O peak is attributed to titanium oxide species and oxygen terminations on the MXene surface, whereas the C–Ti–(OH)*_x_* component originates from hydroxy-terminated surface groups. The high-binding-energy O=C–OH peak corresponds to carboxyl-containing oxygen species. These characteristic MXene surface-termination species were similarly not resolvable in the physical mixture control, further supporting improved surface exposure of the outer, oxygen-terminated Ti-containing surface layer of the d-MXene in the assembled nanoarchitecture. An F 1s spectrum was not acquired in this study. As HF etching is well known to introduce residual surface fluorine terminations on Ti_3_C_2_T*_x_* MXene, F 1s analysis would be expected to provide further insight into the surface termination profile of the d-MXene component; however, its acquisition as a valuable direction for future characterization of this system.

Taken together, the XPS data indicate that both graphene and d-MXene retain their native chemical bonding environments following assembly, with no evidence of new covalent bond formation between the two components. Instead, interfacial assembly is proposed to be driven predominantly by non-covalent interactions, including van der Waals forces and hydrogen bonding between oxygen-containing surface terminations of d-MXene and residual oxygen functionalities of graphene, promoted by repeated nanosheet collisions during ultrasonication. We note that this interpretation relies on the C 1s C–Ti component and complementary structural evidence (PXRD, Raman, and HRTEM/SAED) for confirmation of carbide retention, as the Ti 2p spectrum in this study predominantly reflects an oxidized/terminated Ti surface environment rather than the underlying carbide core directly. Standalone high-resolution XPS spectra of pristine graphene and pristine d-MXene individually were not acquired in this study; comparative confirmation of the individual components relative to the nanoarchitecture is instead provided by PXRD, Raman, HRTEM/SAED, and EDS (Figures 2–4), together with a composition-matched physical mixture control examined by XPS (Supporting Information File 1, Figure S3).

Further supporting evidence is provided by high-resolution XPS comparison between the nanoarchitecture (Figure 5) and the physical mixture control (Supporting Information File 1, Figure S3). While the C 1s C–Ti component appears at a comparable binding energy in both samples (282.4 eV for the physical mixture vs 282.5 eV for the nanoarchitecture), confirming retention of the native d-MXene carbide framework, a clear difference is observed in the Ti 2p and O 1s regions: The physical mixture shows a poorly resolved, high-noise Ti 2p signal with no clearly identifiable surface-termination components in O 1s, whereas the nanoarchitecture exhibits a well-resolved Ti 2p_3/2_/Ti 2p_1/2_ doublet and three distinct O 1s components (Ti–O, C–Ti–(OH)*_x_*, O=C–OH) characteristic of MXene surface terminations. Given the comparable graphene/d-MXene ratio in both samples, this indicates improved surface accessibility of the outer, oxygen-terminated Ti-containing surface layer of the d-MXene following ultrasonication-assisted assembly, consistent with the PXRD and Raman evidence for suppressed nanosheet restacking, rather than reflecting a difference in composition ratio.

### Zeta potential and dynamic light scattering analysis

**Figure 6:**
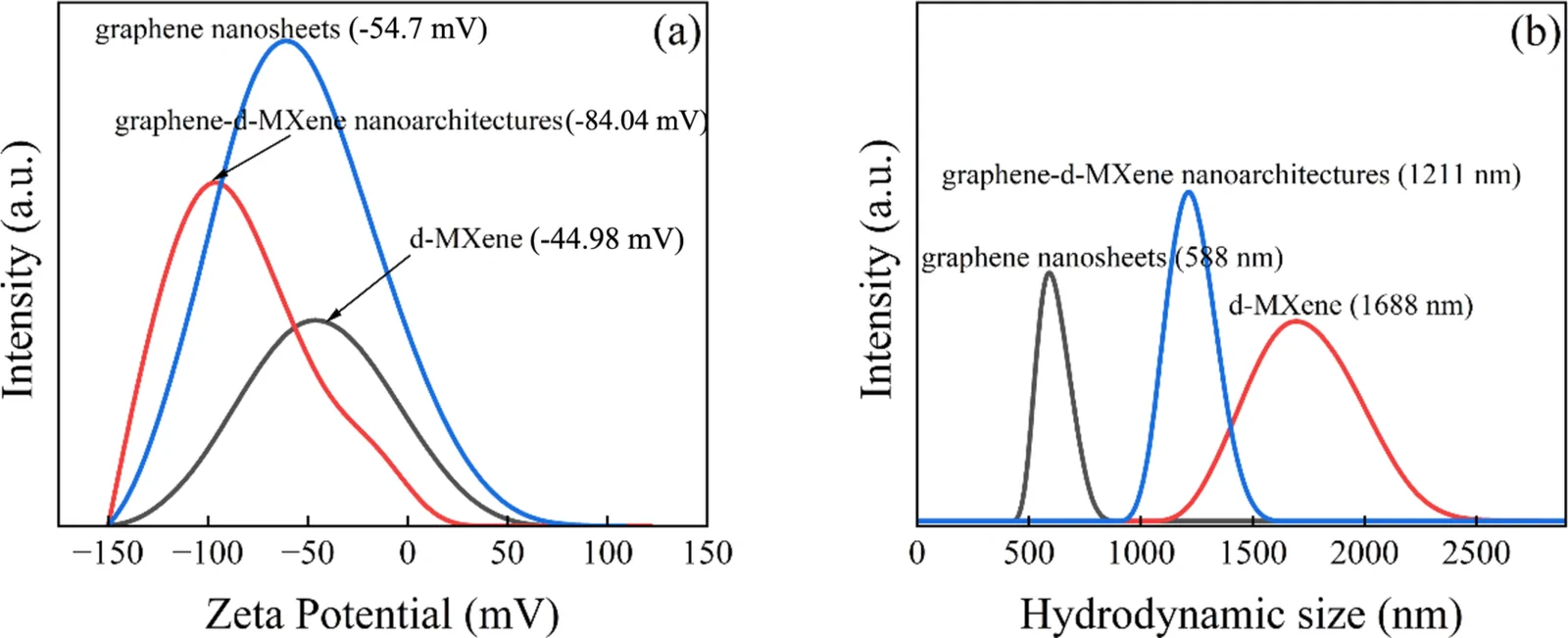
(a) Zeta potential and (b) hydrodynamic size, of graphene nanosheets, d-MXene, and graphene-decorated d-MXene nanoarchitectures.

Zeta potential is a key physicochemical parameter used to evaluate the surface charge and colloidal stability of dispersed nanomaterials. Generally, highly negative zeta potential values indicate strong electrostatic repulsive interactions among suspended particles, thereby suppressing aggregation and improving dispersion stability in colloidal systems. Figure 6a presents the zeta-potential distribution curves of graphene nanosheets, d-MXene, and graphene-decorated d-MXene nanoarchitectures measured by electrophoretic light scattering (ELS) under identical dispersion conditions.

Graphene nanosheets exhibited a zeta potential of −54.7 mV, whereas d-MXene exhibited a value of −44.98 mV. The graphene-decorated d-MXene nanoarchitecture exhibited the most negative zeta potential (−84.04 mV), indicating enhanced electrostatic stabilization compared with the individual components (Table 2).

**Table 2:** Zeta potential values of graphene nanosheets, d-MXene, and graphene-decorated d-MXene nanoarchitectures measured using electrophoretic light scattering.

Sample	Zeta potential (mV)

graphene nanosheets	−54.7
d-MXene	−44.98
graphene-decorated d-MXene nano architectures	−84.04

The substantially more negative zeta potential of the nanoarchitecture relative to either individual component is attributed to two combined effects: (i) graphene-mediated suppression of d-MXene restacking, which increases the exposed surface area and accessibility of negatively charged oxygen-containing functional groups (–O, –OH) on the d-MXene surface that would otherwise remain buried between restacked layers, and (ii) the combined contribution of graphene’s own negatively charged, defect-rich surface groups alongside these newly accessible d-MXene terminations, together producing a net surface charge more negative than either component measured individually. These exposed functional groups increase the net negative surface charge, resulting in stronger electrostatic repulsion between dispersed nanosheets. The increased electrostatic repulsion effectively suppresses nanosheet agglomeration and restacking, thereby improving dispersion homogeneity and colloidal stability. The improved colloidal stability is expected to facilitate more uniform dispersion of the nanoarchitecture in aqueous media, thereby increasing the accessibility of exposed nanosheet surfaces and promoting more effective contact with bacterial cell membranes during antibacterial evaluation. All zeta-potential measurements were performed under identical experimental conditions, including dispersion medium, sample concentration, pH, and temperature, to enable direct comparison among the investigated samples.

It is acknowledged that the zeta potential is sensitive to experimental parameters such as pH, ionic strength, sample concentration, and dispersion medium; while all measurements in this study were performed under identical, fixed conditions to ensure valid comparison between samples, replicate measurements with statistical error analysis (e.g., mean ± standard deviation across independent runs) were not performed in the present study. We acknowledge this as a limitation of the current dataset and recommend replicate zeta-potential measurements with error reporting in future studies to further strengthen the statistical robustness of this comparison.

Complementary dynamic light scattering (DLS) measurements were performed to assess the hydrodynamic size and dispersion behaviour of graphene nanosheets, d-MXene, and the graphene-decorated d-MXene nanoarchitecture in the same aqueous medium (Figure 6b). Graphene nanosheets exhibited the smallest average hydrodynamic size (588 nm), consistent with their comparatively thin, well-dispersed sheet morphology, whereas pristine d-MXene exhibited a substantially larger hydrodynamic size (1658 nm). Following graphene incorporation, the nanoarchitecture exhibited a reduced average hydrodynamic size of 1211 nm relative to pristine d-MXene, in agreement with the zeta-potential trend described above.

It is noted that DLS measures the hydrodynamic diameter of dispersed particles based on their Brownian motion, rather than their true lateral dimensions or thickness. Consequently, for anisotropic two-dimensional nanosheets such as graphene and d-MXene, the measured hydrodynamic size is influenced by sheet geometry, orientation, and possible inter-sheet interactions in suspension, and does not directly correspond to the actual lateral size or thickness of individual nanosheets. Accordingly, the reduction in hydrodynamic size observed by DLS should not, on its own, be taken as direct proof of reduced nanosheet restacking; rather, it is interpreted as a comparative, solution-phase indicator of overall aggregate size among the three samples. Direct structural evidence for suppressed d-MXene restacking is instead provided independently by SEM and HRTEM analysis: SEM imaging (Figure 1c) reveals a less compact, more expanded hierarchical arrangement in the nanoarchitecture relative to the dense, accordion-like stacking of pristine d-MXene (Figure 1b), while HRTEM imaging of the nanoarchitecture (Figure 3g) shows a more open layered architecture with graphene distributed both over and between d-MXene layers, consistent with suppressed restacking. The DLS trend reported here is therefore presented as consistent with, rather than independent proof of, the restacking suppression established through this direct imaging evidence.

### Mechanism of graphene–d-MXene interfacial assembly

Taken together, the structural, spectroscopic, and colloidal characterization presented above converges on a consistent interfacial assembly mechanism. Given that the synthesis involves only ultrasonication of graphene and d-MXene in aqueous suspension, without covalent linking agents or chemical functionalization steps, the interaction between the two components is proposed to be predominantly non-covalent, arising from van der Waals forces and hydrogen bonding between the oxygen-containing surface terminations of d-MXene (–O, –OH) and residual oxygen-containing functional groups on graphene, promoted by repeated nanosheet collisions during ultrasonication.

This mechanism is supported by convergent evidence across all characterization techniques presented above: SEM (Figure 1c) and SEM-EDS elemental mapping (Figure 2d–h) show graphene nanosheets distributed both over and between d-MXene layers with a homogeneous spatial distribution of C, Ti, and O, indicating close-range association without elemental segregation; HRTEM and SAED (Figure 3g–i) directly visualize intimate nanoscale contact between graphene and d-MXene while both lattices retain their individual crystalline identity, rather than merging into a new phase; PXRD (Figure 4a) shows a sharper graphitic (002) reflection in the nanoarchitecture relative to a composition-matched physical mixture; Raman spectroscopy (Figure 4b) shows systematic shifts in the graphene D/G bands and the Ti-related MXene mode, together with a reduced *I*_D_/*I*_G_ ratio relative to the same physical mixture; XPS (Figure 5) shows unchanged C 1s and Ti 2p binding energies relative to the physical mixture control, indicating no new covalent bond formation; and zeta potential (Table 2) shows a substantially more negative surface charge for the nanoarchitecture than either individual component, consistent with electrostatic stabilization from increased exposure of oxygen-containing surface groups. Together, this evidence is consistent with intimate, non-covalent interfacial contact rather than covalent linkage or a specifically “anchored” chemical bond, and the manuscript’s terminology has been revised accordingly throughout.

### Concentration-dependent antibacterial behaviour

#### CFU analysis

**Figure 7:**
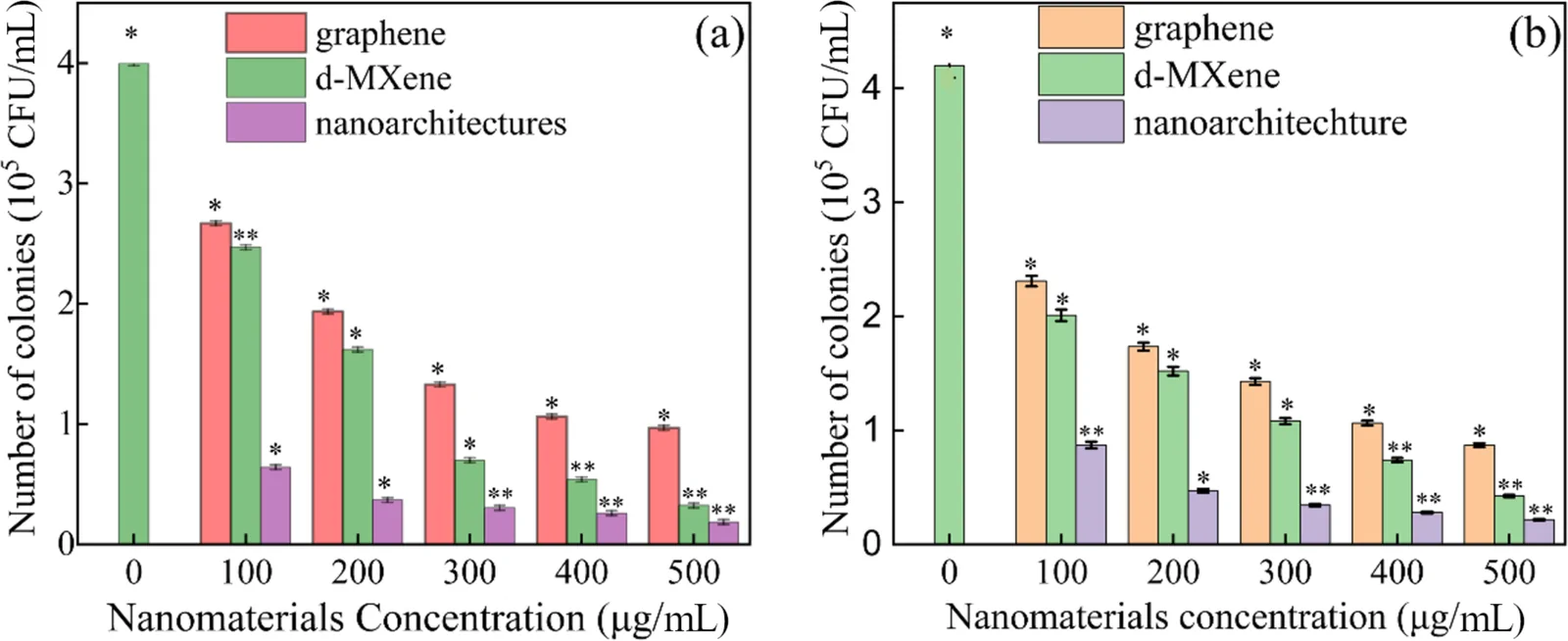
Concentration-dependent antibacterial activity against (a) *E. coli* and (b) *B. cereus* expressed as CFU/mL before and after exposure to graphene nanosheets, d-MXene, and graphene-decorated d-MXene nanoarchitectures at 100–500 µg/mL. Data are presented as mean ± SD (*n* = 3 independent experiments).

The antibacterial performance at different concentrations of graphene nanosheets, d-MXene, and the graphene-decorated d-MXene nanoarchitectures are evaluated against *E. coli* and *B. cereus* using an antimicrobial susceptibility assay by counting bacterial CFUs. Figure 7a,b shows the viable colony counts (CFU/mL) after exposure to graphene nanosheets, d-MXene, and graphene-decorated d-MXene nanoarchitectures at various concentrations (100–500 µg/mL), while the respective values are listed in Table 3. For graphene nanosheets, the bacterial count decreases progressively from 2.67 × 10^5^ CFU/mL (100 µg/mL) to 9.60 × 10^4^ CFU/mL (500 µg/mL), with intermediate values of 2.28 × 10^5^, 1.32 × 10^5^, and 1.06 × 10^5^ CFU/mL at 200, 300 and 400 µg/mL, respectively.

**Table 3:** Concentration-dependent antibacterial activity of graphene, d-MXene, and nanoarchitectures against *E. coli* and *B. cereus* (×10^5^ CFU/mL).

Bacteria	Samples	Concentration of samples (µg/mL)^a^					

		Control	100	200	300	400	500

*E. coli*	graphene	4.00 ± 0.05	2.67 ± 0.07	2.28 ± 0.06	1.32 ± 0.05	1.06 ± 0.03	0.96 ± 0.05
	d-MXene		2.47 ± 0.05	1.62 ± 0.07	0.69 ± 0.05	0.53 ± 0.04	0.32 ± 0.05
	nanoarchitectures		0.64 ± 0.06	0.37 ± 0.05	0.3 ± 0.07	0.26 ± 0.05	0.18 ± 0.04

*B. cereus*	graphene	4.20 ± 0.05	2.31 ± 0.07	1.74 ± 0.06	1.43 ± 0.05	1.06 ± 0.03	0.87 ± 0.05
	d-MXene		2.01 ± 0.05	1.52 ± 0.07	1.08 ± 0.05	0.74 ± 0.04	0.42 ± 0.05
	nanoarchitectures		0.87 ± 0.06	0.47 ± 0.05	0.34 ± 0.07	0.28 ± 0.05	0.21 ± 0.04

^a^Values are expressed as mean ± SD (*n* = 3 independent experiments). Statistical significance was determined using two-way ANOVA followed by Tukey’s post hoc test (*p* < 0.05).

This gradual decline indicates moderate antibacterial activity, likely governed by physical interactions such as membrane damage induced by graphene sheets. d-MXene (MXene) exhibits a stronger antibacterial effect across the entire concentration range. The CFU values decrease from 2.47 × 10^5^ CFU/mL (100 µg/mL) to 3.20 × 10^4^ CFU/mL (500 µg/mL), passing through 1.62 × 10^5^, 6.90 × 10^4^, and 5.30 × 10^4^ CFU/mL at intermediate concentrations. Compared to graphene nanosheets, MXene consistently shows lower bacterial counts at each concentration, indicating its higher intrinsic antibacterial activity, which can be attributed to its surface terminations and enhanced chemical reactivity. All materials exhibited concentration-dependent antibacterial activity; however, the nanoarchitecture consistently produced the greatest reduction in bacterial viability over the entire concentration range. The graphene-decorated d-MXene nanoarchitectures has observed lowest CFU values at all concentrations, clearly outperforming both individual components. The bacterial count decreases from 6.40 × 10^4^ CFU/mL (100 µg/mL) to 1.80 × 10^4^ CFU/mL (500 µg/mL), with intermediate values of 3.70 × 10^4^, 3.00 × 10^4^, and 2.60 × 10^4^ CFU/mL. At 500 µg/mL, the nanoarchitectures achieves the lowest count (1.80 × 10^4^ CFU/mL), which is a fifth of that of graphene nanosheets and half of that of d-MXene, confirming its highest antibacterial efficiency.

A similar concentration-dependent antibacterial behaviour was observed against the Gram-positive bacterium *B. cereus* (Figure 7b). The untreated control exhibited a viable bacterial count of 4.20 × 10^5^ CFU/mL. Following treatment with graphene nanosheets, the colony count decreased from 2.31 × 10^5^ CFU/mL at 100 µg/mL to 8.70 × 10^4^ CFU/mL at 500 µg/mL, with intermediate values of 1.74 × 10^5^, 1.43 × 10^5^, and 1.06 × 10^5^ CFU/mL at 200, 300, and 400 µg/mL, respectively. d-MXene exhibited enhanced antibacterial activity, reducing the viable bacterial count from 2.01 × 10^5^ CFU/mL at 100 CFU/mL to 4.20 × 10⁴ CFU/mL at 500 µg/mL, with intermediate values of 1.52 × 10^5^, 1.08 × 10^5^, and 7.40 × 10^4^ CFU/mL. The graphene-decorated d-MXene nanoarchitectures again demonstrated the strongest antibacterial performance, decreasing the bacterial count from 8.70 × 10^4^ CFU/mL at 100 µg/mL to only 2.10 × 10^4^ CFU/mL at 500 µg/mL, with intermediate values of 4.70 × 10^4^, 3.40 × 10^4^, and 2.80 × 10^4^ CFU/mL. At the highest concentration, the nanoarchitectures produced a fourfold lower viable bacterial count than graphene nanosheets and nearly twofold lower than d-MXene, demonstrating effective growth suppression of both Gram-negative and Gram-positive bacteria.

Overall, the antibacterial performance against both *E. coli* and *S. aureus* follows the order: graphene nanosheets < d-MXene < graphene-decorated d-MXene nanoarchitectures, across all investigated concentrations. The superior performance of the nanoarchitectures is attributed to the non-covalent interfacial interaction between graphene nanosheets and MXene within a graphene-decorated d-MXene nanoarchitectures.

Two-way ANOVA followed by Tukey’s post hoc multiple comparison test demonstrated that bacterial inhibition was significantly influenced by both material type and nanomaterial concentration (*p* < 0.05). The graphene-decorated d-MXene nanoarchitectures consistently exhibited significantly enhanced antibacterial activity than graphene nanosheets and d-MXene against both *E. coli* and *B. cereus* across the investigated concentration range.

#### OD_600_ growth analysis

**Figure 8:**
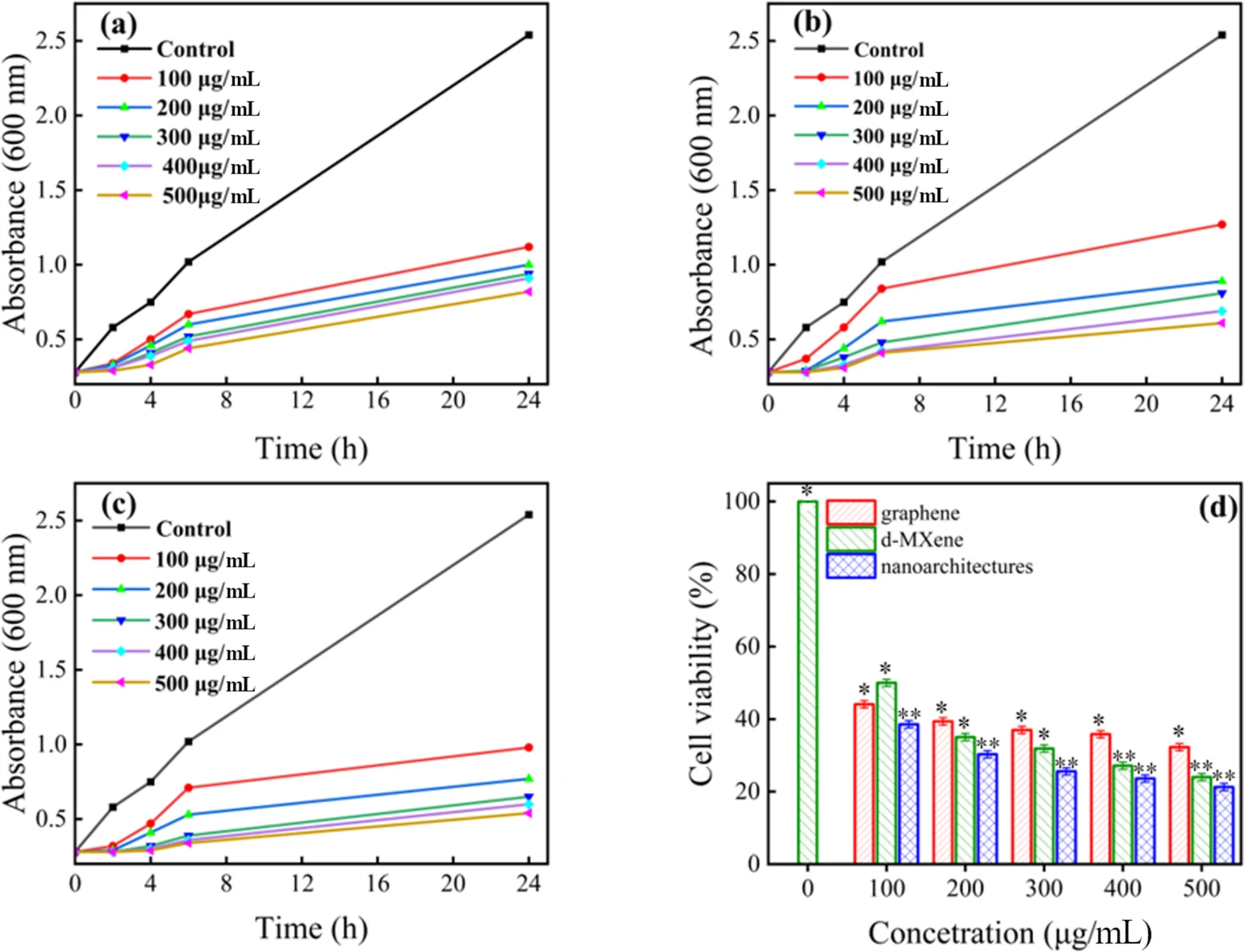
Time-dependent OD_600_ growth curves of *E. coli* treated with (a) graphene nanosheets, (b) d-MXene, and (c) graphene-decorated d-MXene nanoarchitectures; (d) concentration-dependent cell viability at concentrations ranging from 100 to 500 µg/mL. Data are presented as mean ± SD (*n* = 3 independent experiments, *p < 0.05, **p < 0.01).

**Figure 9:**
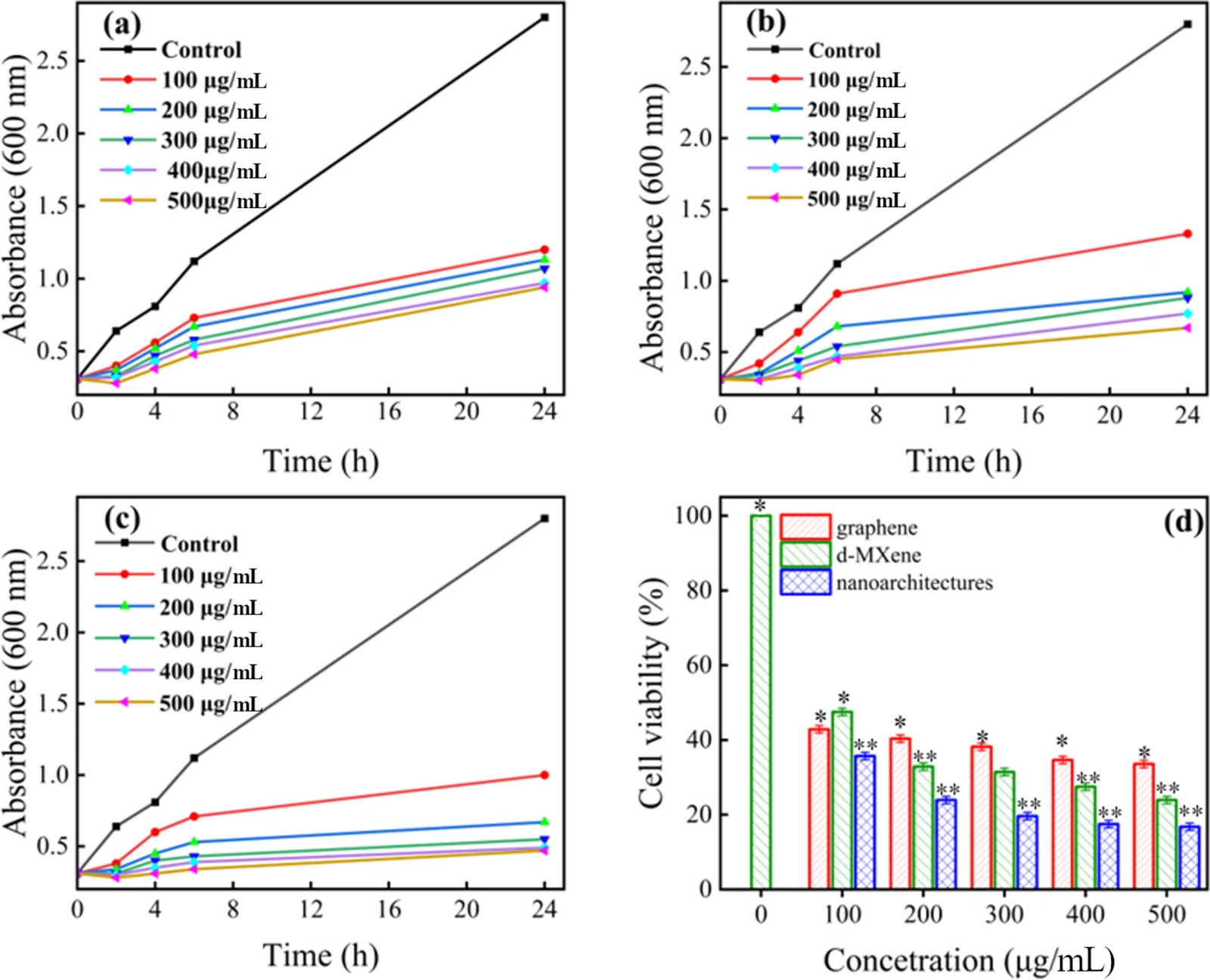
Time-dependent OD600 growth curves of *B. cereus* treated with (a) graphene nanosheets, (b) d-MXene, and (c) graphene-decorated d-MXene nanoarchitectures; (d) concentration-dependent cell viability at concentrations ranging from 100 to 500 µg/mL. Data are presented as mean ± SD (*n* = 3 independent experiments, *p < 0.05, **p < 0.01).

The antibacterial performance of graphene nanosheets, d-MXene, and the graphene-decorated d-MXene nanoarchitectures is further evaluated through a broth microdilution assay by determining optical density at 600 nm (OD_600_), monitoring bacterial growth kinetics in liquid culture. Figure 8a–c illustrates the time-dependent growth behaviour of *E. coli* treated with graphene nanosheets, d-MXene, and graphene-decorated d-MXene nanoarchitecture, respectively, at concentrations from 100–500 µg/mL, whereas Figure 9a–c presents the corresponding OD_600_ growth curves for *B. cereus*.

All experiments started with identical initial bacterial densities, confirming uniform initial bacterial loading. In the absence of nanomaterials (control), *E. coli* exhibited rapid exponential growth, with the OD_600_ increasing continuously throughout the incubation period and reaching the highest value after 24 h indicating unrestricted bacterial proliferation in nutrient-rich medium. In contrast, cultures treated with graphene nanosheets, d-MXene, and graphene-decorated d-MXene nanoarchitectures displayed significant growth suppression at all time intervals, which intensified systematically with increasing concentration and prolonged exposure.

For graphene nanosheets (Figure 8a), the OD_600_ values at 2 h ranged from 0.34 to 0.29 as the concentration increased from 100 to 500 µg/mL, followed by further reductions at 4 h (0.50 to 0.33) and 6 h (0.67 to 0.44), indicating a clear concentration-dependent retardation of bacterial growth. After 24 h of incubation, the OD_600_ decreased from 1.12 at 100 µg/mL to 0.82 at 500 µg/mL, demonstrating a moderate but distinct antibacterial effect of graphene nanosheets. In contrast, d-MXene (Figure 8b) exhibited stronger inhibition throughout the incubation period, with OD_600_ values at 2 h decreasing from 0.37 to 0.28 and at 6 h from 0.84 to 0.41 as the concentration was increased, reflecting more effective early-stage growth suppression. After 24 h, OD_600_ values further declined from 1.27 with 100 µg/mL to 0.61 with 500 µg/mL of d-MXene, highlighting the superior antibacterial activity of MXene nanosheets compared to graphene nanosheets. Notably, the graphene-decorated d-MXene nanoarchitectures (Figure 8c) showed the most pronounced growth inhibition across all concentrations and time points. Even at early incubation times, OD_600_ values remained consistently lower than those of the individual components, and after 24 h, the OD_600_ decreased from 0.98 with 100 µg/mL to as low as 0.54 with 500 µg/mL of graphene-decorated d-MXene nanoarchitectures, while at higher concentrations the difference becomes more pronounced. The growth curves also appear flatter compared to the individual materials, indicating more effective suppression of bacterial proliferation over time. A direct comparison across all concentrations clearly demonstrates the superior performance of the nanoarchitectures. At 100 µg/mL, the OD_600_ value for the nanoarchitectures (0.95) is lower than that for graphene nanosheets (1.10) and MXene (1.25), indicating stronger inhibition at low dosage. This trend continues at higher concentrations; at 500 µg/mL, the nanoarchitectures (0.55) inhibit bacterial growth stronger than graphene nanosheets (0.80) and slightly stronger than d-MXene (0.60).

A similar concentration-dependent antibacterial behaviour was observed for the Gram-positive bacterium *B. cereus*, as illustrated in Figure 9a–c. The untreated control exhibited rapid bacterial proliferation, with the OD_600_ increasing from 0.31 at 0 h to 0.64 at 2 h, 0.81 at 4 h, 1.12 at 6 h, and finally reaching 2.80 after 24 h. In comparison, all nanomaterial-treated cultures showed significant suppression of bacterial growth, which became progressively stronger with increasing concentration. For graphene nanosheets (Figure 9a), the OD_600_ values at 24 h gradually decreased from 1.20 at 100 µg/mL to 0.94 at 500 µg/mL, with corresponding values of 0.40–0.26, 0.56–0.38, and 0.73–0.48 at 2, 4, and 6 h, respectively. The d-MXene sample (Figure 9b) exhibited stronger antibacterial activity, reducing the 24 h OD_600_ absorbance from 1.33 at 100 µg/mL to 0.67 at 500 µg/mL, accompanied by lower OD_600_ values throughout the incubation period compared with graphene nanosheets. Among all investigated materials, the graphene-decorated d-MXene nanoarchitectures (Figure 9c) exhibited the most pronounced inhibition of *B. cereus* growth. The OD_600_ values continuously decreased from 0.99 at 100 µg/mL to only 0.47 at 500 µg/mL after 24 h, while the early-stage OD_600_ values also remained consistently lower than those of graphene nanosheets and d-MXene.

Overall, the inhibition kinetics of both *E. coli* and *B. cereus* consistently demonstrate that the antibacterial performance follows the order: graphene nanosheets < d-MXene < graphene-decorated d-MXene nanoarchitectures. This trend is in excellent agreement with the CFU assay, confirming that the graphene-decorated d-MXene nanoarchitectures exhibit the strongest bacteriostatic effect against both Gram-negative and Gram-positive bacteria. Two-way ANOVA followed by Tukey’s post hoc multiple comparison test confirmed statistically significant suppression of bacterial growth with increasing nanomaterial concentration and material type (*p* < 0.05). The graphene-decorated d-MXene nanoarchitectures exhibited significantly stronger growth inhibition than pristine graphene nanosheets and d-MXene against both *E. coli* and *Bacillus cereus* (*p* < 0.05), demonstrating the effectiveness of the nanoarchitecture in broad-spectrum antibacterial applications.

#### Cell viability analysis

The antibacterial performance of graphene nanosheets, d-MXene, and the graphene-decorated d-MXene nanoarchitectures was also evaluated as cell viability after 24 h of incubation against *E. coli* and *B. cereus* (Figure 8d, Figure 9d and Table 4). The previously reported antimicrobial activities summarized in Table 5 further highlight the significance and effectiveness of the present nanoarchitecture. For *E. coli* (Figure 8d) a concentration-dependent decrease in cell viability is observed for graphene nanosheets, d-MXene, and the graphene-decorated d-MXene nanoarchitectures; however, the nanoarchitectures consistently demonstrates the superior antibacterial efficiency across the entire concentration range.

**Table 4:** Concentration-dependent cell viability (%) of *E. coli* after treatment with graphene nanosheets, d-MXene, and graphene-decorated d-MXene nanoarchitectures.

Bacterium	Nanomaterials	Concentration of samples (µg/mL) and cell viability (%)^a^				

		100	200	300	400	500

*E. coli*	graphene nanosheets	44.09 ± 0.9	39.37 ± 0.8	37.00 ± 0.9	35.82 ± 0.7	32.28 ± 0.8
	d-MXene	50 ± 0.8	35.04 ± 1.0	31.88 ± 0.7	27.16 ± 0.9	24.01 ± 0.8
	nanoarchitectures	38.58 ± 0.7	30.31 ± 0.9	25.59 ± 0.8	23.62 ± 0.9	21.25 ± 0.7

*B. cereus*	graphene nanosheets	42.85 ± 0.9	40.35 ± 0.8	38.21 ± 0.9	34.64 ± 0.7	33.57 ± 0.8
	d-MXene	47.8 ± 0.8	32.85 ± 1.0	31.42 ± 0.7	27.5 ± 0.9	23.92 ± 0.8
	nanoarchitectures	35.71 ± 0.7	23.92 ± 0.9	19.64 ± 0.8	17.5 ± 0.9	16.78 ± 0.7

^a^Values are expressed as mean ± SD (*n* = 3 independent experiments). Statistical significance was determined using two-way ANOVA followed by Tukey’s post hoc test (*p* < 0.05).

At 100 µg/mL, graphene nanosheets, d-MXene, and the nanoarchitectures exhibited cell viabilities of 44.09 ± 0.90%, 50.00 ± 0.80%, and 38.58 ± 0.70%, respectively. Increasing the concentration to 200 µg/mL further reduced the bacterial viability to 39.37 ± 0.80%, 35.04 ± 1.00%, and 30.31 ± 0.90%, respectively. At 300 µg/mL, the corresponding cell viabilities decreased to 37.00 ± 0.90%, 31.88 ± 0.70%, and 25.59 ± 0.80%, while at 400 µg/mL they further declined to 35.82 ± 0.70%, 27.16 ± 0.90%, and 23.62 ± 0.90%, respectively. At the highest concentration (500 µg/mL), graphene nanosheets, d-MXene, and the graphene-decorated d-MXene nanoarchitectures exhibited minimum cell viabilities of 32.28 ± 0.80%, 24.01 ± 0.80%, and 21.25 ± 0.70%, respectively. Compared with graphene nanosheets and d-MXene, the nanoarchitectures reduced the bacterial viability by approximately 34% and 11.5%, respectively, confirming its enhanced antibacterial efficacy against Gram-negative bacteria. A similar concentration-dependent reduction in bacterial viability was observed for the Gram-positive bacterium *B. cereus* (Figure 9d and Table 4). Graphene nanosheets exhibited cell viabilities of 42.85 ± 0.90%, 40.35 ± 0.80%, 38.21 ± 0.90%, 34.64 ± 0.70%, and 33.57 ± 0.80% at concentrations of 100, 200, 300, 400, and 500 µg/mL, respectively. Under identical conditions, d-MXene produced lower bacterial viability values of 47.80 ± 0.80%, 32.85 ± 1.00%, 31.42 ± 0.70%, 27.50 ± 0.90%, and 23.92 ± 0.80%, indicating stronger antibacterial activity than graphene nanosheets. Among all investigated materials, the graphene-decorated d-MXene nanoarchitectures again exhibited the highest antibacterial performance, reducing the bacterial viability from 35.71 ± 0.70% at 100 µg/mL to only 16.78 ± 0.70% at 500 µg/mL, with intermediate values of 23.92 ± 0.90%, 19.64 ± 0.80%, and 17.50 ± 0.90% at 200 µg/mL, 300 µg/mL, and 400 µg/mL, respectively. At the highest concentration, the nanoarchitectures decreased the bacterial viability by 50% compared with graphene nanosheets and about 30% compared with d-MXene, demonstrating its remarkable antibacterial efficacy against Gram-positive bacteria. The consistently lower cell viability values indicate that the nanoarchitecture effectively inhibits bacterial survival regardless of bacterial cell-wall structure.

Overall, the cell viability results for both *E. coli* and *B. cereus* demonstrate that the antibacterial performance follows the order: graphene nanosheets > d-MXene > graphene-decorated d-MXene nanoarchitectures, where lower bacterial cell viability corresponds to stronger antibacterial activity. This trend is fully consistent with the OD_600_ growth kinetics and CFU analyses, confirming that the graphene-decorated d-MXene nanoarchitectures possess the highest bactericidal efficiency among the investigated materials owing to the nanoarchitecture which enhances bacterial membrane disruption and oxidative stress. Two-way ANOVA followed by Tukey’s post hoc multiple comparison test demonstrated that bacterial viability was significantly affected by both material type and nanomaterial concentration (*p* < 0.05). The graphene-decorated d-MXene nanoarchitectures exhibited significantly lower bacterial cell viability than graphene nanosheets and d-MXene against both *E. coli* and *Bacillus cereus* across the investigated concentration range (*p* < 0.05), confirming that both nanomaterial composition and concentration significantly influence antibacterial performance.

A comparison with literature (Table 5) underscores the enhanced performance and competitive edge of the present study. For instance, graphene showed cell viability values of 35.2% and 65.33%, and G-Fe_3_O_4_ composites showed values of 22.66%; reduced graphene oxide (rGO)/Ag exhibited a higher viability of 25.8%. Similarly, Ti_3_CNT*_x_* and its modified forms, such as Ag/Ti_3_CNT*_x_* (38%) and POSS-doped Ti_3_C_2_T*_x_* (41.68%), demonstrate inferior antibacterial activity compared to the present system. Notably, in this work, the graphene-decorated d-MXene nanoarchitectures (21.25%) exhibits lower cell viability than all reported counterparts, including pristine graphene nanosheets (32.28%) and d-MXene (24.01%) for *E. coli* and 16.78%, 32.57% and 23.92% for *B. cereus* respectively.

**Table 5:** The percentage cell viability against *E. coli* and *B. cereus*.

Nanomaterial	Bacterium	Concentration	Time	Cell viability	Assay/method	Ref.

graphene	*E. coli*	200 ppm	1.5 h	65.33%	viability after plating	[36]
rGO-Ag	*E. coli*	–	4 h	25.8%	cell viability	[37]
G-Fe_3_O_4_	*E. coli*	200 ppm	1.5 h	22.66%	time-dependent viability assay	[36]
Ti_3_CNT*_x_*	*E. coli*	250 mg L^−1^	72 h	50%	OD-based viability	[38]
Ag/Ti_3_CNT*_x_*	*E. coli*	250 mg L^−1^	72 h	38%	OD-based viability	[38]
POSS-doped Ti_3_C_2_T*_x_*	*E. coli*	PMPs films	24 h	41.68%	bacterial viability/CFU	[39]
ZnO NPs	*B. cereus*	0.8 mg/mL	24 h	57.64%	cell viability	[40]
graphene nanosheets	*E. coli*	500 µg/mL	24 h	32.28%	cell viability	this work
d-MXene	*E. coli*	500 µg/mL	24 h	24.01 ± 0.8%	cell viability	this work
nanoarchitecture	*E. coli*	500 µg/mL	24 h	21.25 ± 0.7%	cell viability	this work
graphene nanosheets	*B. cereus*	500 µg/mL	24 h	32.57 ± 0.8%	cell viability	this work
d-MXene	*B. cereus*	500 µg/mL	24 h	23.92 ± 0.8%	cell viability	this work
nanoarchitecture	*B. cereus*	500 µg/mL	24 h	16.78 ± 0.7%	cell viability	this work

This clearly demonstrates that the graphene-decorated d-MXene nanoarchitecture not only outperforms its individual constituents but also surpasses a wide range of previously reported nanomaterials, establishing it as a highly effective and competitive candidate for advanced antimicrobial applications. It should be noted, however, that the literature studies summarized in Table 5 were conducted under different experimental conditions, including variations in bacterial strains, nanomaterial synthesis methods, concentrations, incubation times, and antibacterial assay protocols. Therefore, the comparison is intended to provide a qualitative benchmark of the antibacterial performance of the present graphene-decorated d-MXene nanoarchitecture rather than a direct quantitative comparison under identical experimental conditions. Nevertheless, the consistently lower bacterial cell viability achieved by the present nanoarchitecture demonstrates its highly competitive antibacterial performance relative to representative graphene- and MXene-based nanomaterials reported in the literature.

#### Superoxide radical detection

**Figure 10:**
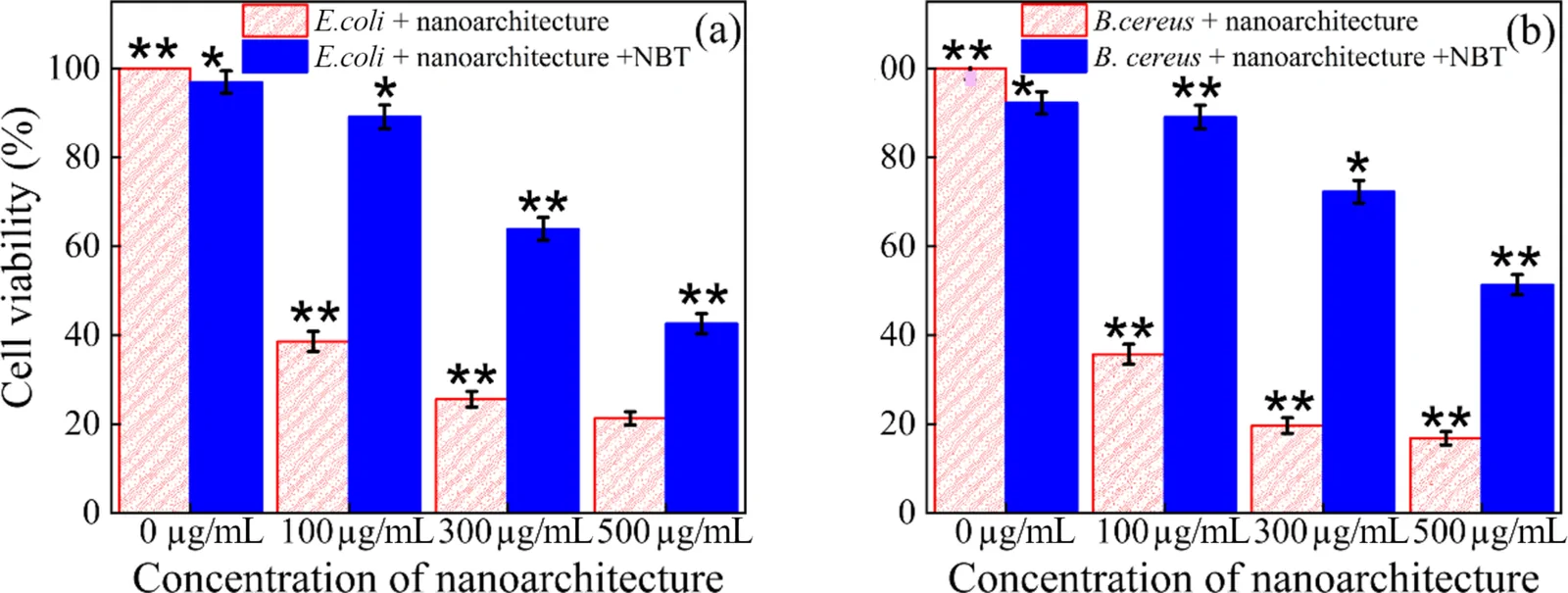
Cell viability of bacterial assay and NBT incubated bacterial assay with control, 100 μg/mL, 300 μg/mL, and 500 μg/mL concentration of graphene-decorated d-MXene nanoarchitecture: (a) *E. coli* and (b) *B. cereus.* Error bars represent the standard deviation of three independent experiments (mean ± SD, *n* = 3, *p < 0.05, **p < 0.01).

The possible contribution of ROS to the antibacterial activity of the nanoarchitecture was evaluated. Specifically, the focus was on superoxide anion radicals (O_2_^•−^). For this, a nitro blue tetrazolium (NBT) inhibition assay was performed. This assay was run alongside the standard optical density (OD_600_)-based cell viability assay. Both assays were tested against *E. coli* and *B. cereus*. The contribution of superoxide-mediated oxidative stress to the antibacterial activity of the graphene-decorated d-MXene nanoarchitecture was assessed by comparing OD_600_-based cell viability of *E. coli* and *B. cereus* in the presence and absence of NBT (Figure 10) [41].

Treatment with the nanoarchitecture alone produced a clear, concentration-dependent decrease in bacterial viability for both strains. For *E. coli*, viability decreased from 100% (control) to 38.58 ± 0.7%, 25.59 ± 0.8%, and 21.25 ± 0.7% at 100, 300, and 500 μg/mL, respectively. A comparable trend was observed for *B. cereus*, with viability declining from 100% (control) to 35.71 ± 0.7%, 19.64 ± 0.8%, and 16.78 ± 0.7% over the same concentration range. Co-incubation with NBT significantly attenuated this antibacterial effect at every concentration tested. For *E. coli*, viability increased to 96.95 ± 0.9%, 89.13 ± 0.8%, 63.91 ± 0.7%, and 42.60 ± 0.5% at 0, 100, 300, and 500 μg/mL, respectively, corresponding to a rescue of approximately 50%, 38%, and 21% at the three treated concentrations. For *B. cereus*, NBT co-treatment increased viability to 92.27 ± 0.7%, 89.09 ± 0.6%, 72.27 ± 0.6%, and 51.36 ± 0.4%, representing a rescue of approximately 53%, 52%, and 34%. Three independent experiments were performed for each condition (*n* = 3; mean ± SD). These results indicate that superoxide radical generation contributes substantially to the observed antibacterial activity of the nanoarchitecture against both Gram-negative and Gram-positive bacteria. Two-way ANOVA followed by Tukey’s post hoc multiple comparison test demonstrated that bacterial inhibition was significantly influenced by both material type and nanomaterial concentration (*p* < 0.05). The graphene-decorated d-MXene nanoarchitectures consistently exhibited significantly enhanced antibacterial activity than graphene nanosheets and d-MXene against both *E. coli* and *B. cereus* across the investigated concentration range.

The partial restoration of bacterial viability upon NBT co-treatment provides indirect evidence that superoxide-mediated oxidative stress contributes to the antibacterial action of the graphene-decorated d-MXene nanoarchitecture. Since NBT selectively intercepts O_2_^•−^ prior to downstream oxidative damage to lipids, proteins, and nucleic acids, the extent of viability rescue offers an approximate estimate of the fraction of toxicity attributable to superoxide-driven pathways at each concentration tested. Notably, complete recovery of viability was not achieved even in the presence of NBT, particularly at the highest nanoarchitecture concentration (500 μg/mL), where residual toxicity remained substantial (43% and 51% viability for *E. coli* and *B. cereus*, respectively, relative to ≈97% and ≈92% for untreated controls). This indicates that ROS generation, while a significant contributor, is not the sole antibacterial mechanism. The unrescued toxicity is consistent with a complementary structural/physical contribution-exposed graphene-edge interaction, suppressed MXene restacking, and defect-enriched interfacial regions arising from the nanoarchitecture assembly-mechanisms widely reported for graphene- and MXene-based nanomaterials, whose sharp nanosheet edges and high surface accessibility can directly compromise bacterial membrane integrity independent of oxidative pathways. The comparatively greater NBT-mediated rescue observed for *B. cereus* relative to *E. coli* at matched concentrations (e.g., 72% versus 64% at 300 μg/mL) may reflect differences in cell envelope architecture between the two strains. The thicker peptidoglycan layer of *B. cereus* may confer partial resistance to direct physical puncture by the nanosheets, rendering ROS-mediated oxidative damage proportionally more important to its overall antibacterial susceptibility relative to the thinner, more readily disrupted outer membrane of *E. coli*.

It should be noted that the present NBT-based comparison reflects indirect chemical-scavenger evidence rather than direct radical detection, and should therefore be interpreted as an approximate estimate rather than a precise mechanistic quantification. The relative contributions of the individual structural factors (edge density, restacking suppression, and interfacial defect density) were not experimentally decoupled in this study as they arise concurrently from the same nanoarchitecture assembly process. Future work incorporating EPR spin-trapping, DCFH-DA fluorescence, or SOD/catalase enzymatic controls would enable more precise resolution of the ROS-specific contribution, while structurally decoupled analogues (e.g., restacked or edge-density-controlled samples) would help isolate the physical/structural contributions. Taken together, these findings support a dual mechanism in which structural membrane disruption and superoxide-mediated oxidative stress act in combination to produce the enhanced antibacterial activity of the graphene-decorated d-MXene nanoarchitecture.

#### Proposed antibacterial mechanism

**Figure 11:**
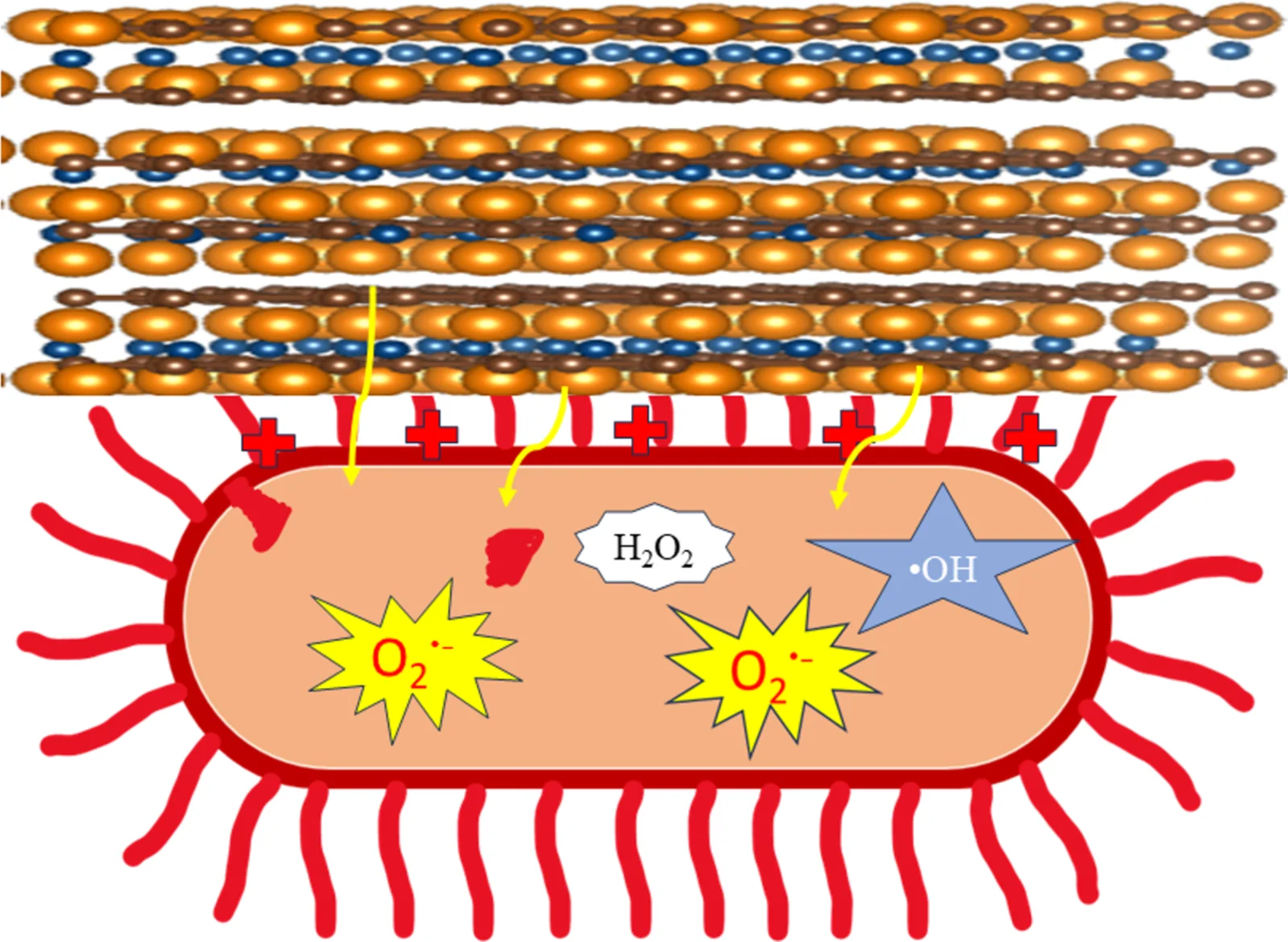
Schematic representation of the cooperative antibacterial mechanism of the graphene-decorated d-MXene nanoarchitecture.

The enhanced antibacterial activity of the graphene-decorated d-MXene nanoarchitecture is primarily associated with the formation of an interfacially assembled 2D/2D layered architecture possessing increased surface accessibility, suppressed nanosheet restacking, and abundant exposed edge-active regions (Figure 11). During ultrasonication-assisted assembly, graphene nanosheets become uniformly integrated with delaminated MXene layers, generating an interconnected nanoarchitecture with improved structural openness and accessible interfacial domains relative to the pristine components. The exposed graphene edges, accessible d-MXene surfaces, and interfacial domains can increase bacteria-nanomaterial contact and promote localized interactions with the bacterial cell envelope, potentially disturbing membrane integrity and impairing bacterial growth.

This structural interpretation is consistent with the concentration-dependent antibacterial trends observed across all three complementary assays. CFU analysis showed a progressive decline in viable bacterial counts with increasing nanoarchitecture concentration for both *E. coli* and *B. cereus*, while OD_600_ growth-kinetics measurements corroborated this trend, showing flatter, more strongly suppressed growth curves for the nanoarchitecture relative to pristine graphene nanosheets or d-MXene alone. The optical density-based cell viability assay (Table 4) further confirmed this pattern, with the nanoarchitecture consistently producing the lowest viability values among the three materials across the full concentration range for both bacterial strains. In addition to these membrane-associated effects, the NBT assay provides evidence for a possible contribution of superoxide-associated oxidative stress to the antibacterial response. The concentration-dependent NBT response observed for the nanoarchitecture is consistent with enhanced superoxide generation during nanomaterial–bacteria interactions, particularly at higher concentrations, for both *E. coli* and *B. cereus*. Comparison of viability with and without NBT co-treatment indicates that superoxide quenching restores a substantial fraction of viability at each tested concentration, suggesting that ROS-associated pathways account for a meaningful share of the overall antibacterial effect; however, because this comparison reflects indirect chemical-scavenger evidence rather than direct radical detection (e.g., via EPR spin-trapping or DCFH-DA fluorescence), it should be interpreted as an approximate estimate rather than a precise mechanistic quantification.

Taken together, the superior antibacterial performance of the graphene-decorated d-MXene nanoarchitecture is attributed to the cooperative contribution of: (i) exposed edge-rich graphene domains capable of direct membrane interaction, (ii) enhanced surface accessibility arising from suppression of MXene restacking, (iii) defect-enriched interfacial regions generated during nanoarchitecture assembly, and (iv) a possible additional contribution from ROS-mediated oxidative stress. It should be noted that the present study was not designed to individually resolve the quantitative contribution of each structural factor (i–iii), as these arise concurrently from the same assembly process and were not experimentally decoupled (e.g., via restacked or edge-density-controlled analogues). Similarly, while the NBT assay provides an approximate estimate of the superoxide-attributable fraction of toxicity, it does not resolve contributions from other potential ROS species. Future studies incorporating EPR spin-trapping, SOD/catalase controls, and structurally decoupled analogues would enable a more precise mechanistic apportionment. Overall, the interconnected 2D/2D nanoarchitecture enhances nanosheet accessibility, colloidal homogeneity, and bacteria-surface contact, thereby providing a structural basis for the superior antibacterial activity observed against both *E. coli* and *B. cereus*, with a plausible secondary contribution from oxidative stress pathways.

## Conclusion

A graphene-decorated d-MXene nanoarchitecture was successfully engineered through a simple, non-toxic bottom-up ultrasonication-assisted interfacial assembly strategy to elucidate the role of nanoarchitecture organization in antibacterial functionality. By integrating graphene nanosheets with d-MXene through controlled interfacial assembly, this strategy effectively addressed the aggregation and restacking limitations of d-MXene while generating an accessible interfacial architecture with enhanced colloidal stability.

These structural and colloidal advantages translated directly into superior, concentration-dependent antibacterial activity against both *E. coli* and *B. cereus* compared with pristine graphene nanosheets and d-MXene under identical experimental conditions, demonstrating that nanoarchitecture formation provides an advantage beyond the intrinsic antibacterial activity of either individual component.

The enhanced antibacterial performance is attributed to the collaborative effects of improved colloidal stabilization, suppressed nanosheet aggregation, increased nanosheet accessibility, and greater exposure of active interfacial domains, which collectively promote more efficient bacteria–nanomaterial interactions. The key advance of this work is therefore the establishment of a structure–property–performance relationship in which interfacial organization and dispersion-state control play important roles in antibacterial enhancement, rather than simple material hybridization alone. The NBT results further suggest a possible contribution from superoxide-associated oxidative stress; however, dedicated mechanistic studies are required to establish its specific contribution to bacterial inactivation. Overall, this study provides a useful design principle for engineering accessible and colloidally stable graphene/MXene nanoarchitectures, establishing a foundation for their further investigation against multidrug-resistant pathogens, biofilms, and practical biomedical or environmental systems.

## Experimental

### Materials

Hydrofluoric acid (HF, 48%), Ti_3_AlC_2_ (98%), and dimethyl sulfoxide (DMSO, 99.9%) were procured from Sigma Aldrich. Graphene nanosheets was purchase from adnano technologies and *E. coli* (ATCC 25923) and *B. cereus* (ATCC 11778) were purchased from Chandigarh, India’s Institute of Microbial Technology (IMTECH). Nitro blue tetrazolium chloride (NBT) was purchased from SRL Pvt. Ltd.

### Synthesis of Ti_3_C_2_T*_x_* and d-MXene

**Figure 12:**
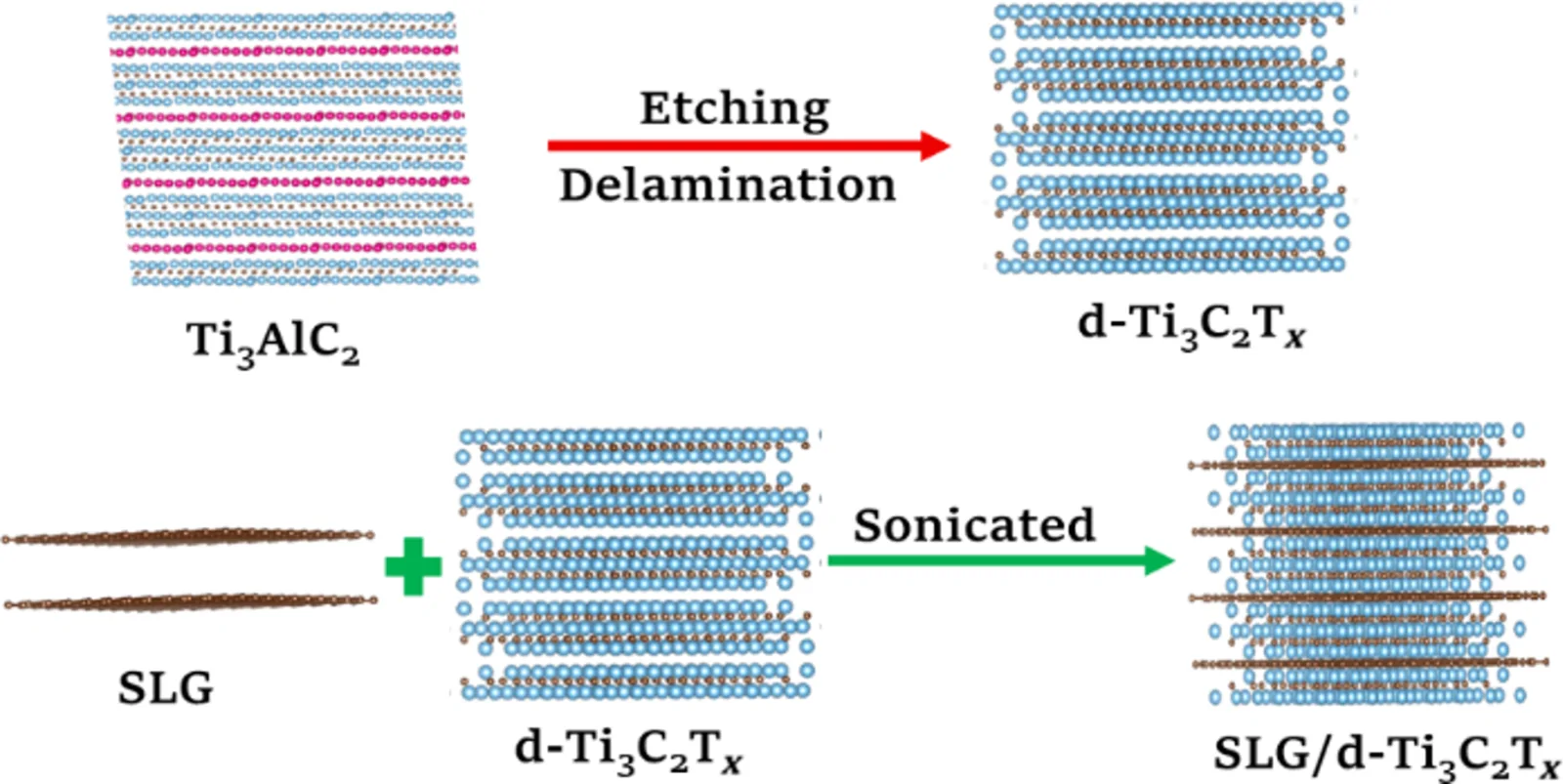
Schematic illustration of the bottom-up assembly of graphene-decorated d-MXene nanoarchitectures (graphene nanosheets/d-MXene).

Pristine Ti_3_C_2_T*_x_* was synthesized by HF etching of Ti_3_AlC_2_. Initially 0.5 g of Ti_3_AlC_2_ was added slowly to avoid the exothermic reaction under stirring at 400 rpm at 40 °C. After 24 h of stirring, the solution was added to 200 mL DI water and washed by centrifugation at 3500 rpm until pH 6. This indicated the full removal of HF from the sample. The obtained precipitate was added in 50 mL DI water and centrifuged at 5000 rpm for 30 min. The obtained precipitate was put to dry for 12 h at 60 °C, yielding a black powder of pristine Ti_3_C_2_T*_x_*. To delaminate, 0.25 g of pristine Ti_3_C_2_T*_x_* was gradually added in 5 mL of DMSO followed by stirring at 400 rpm for 24 h at 40 °C. After stirring, the solution was added to 200 mL DI water and washed by centrifugation at 3500 rpm until pH 6. The obtained precipitate was dispersed in DI water and sonicated for 60 min by a probe sonicator at 50% amplitude and 500 Watt power with 3 s on and 2 s off steps. After filtration and drying at 60 °C for 12 h, d-Ti_3_C_2_T*_x_* was obtained as dark black powder. Figure 12 show the synthesis scheme of d-MXene.

### Assembly of graphene-decorated d-MXene nanoarchitectures

To prepare the graphene-decorated d-MXene nanoarchitectures (graphene nanosheets/d-MXene) 0.050 g of graphene nanosheets was dispersed in 200 mL of DI water and ultrasonicated for 2 h. Afterward, 0.005 g of d-Ti_3_C_2_T*_x_* was gradually introduced into the dispersion, followed by an additional 2 h of ultrasonication. The suspension was then centrifuged twice at 10,000 rpm for 30 min. Finally, the collected precipitate was dried at 60 °C for 24 h. The synthesis scheme of graphene-decorated d-MXene nanoarchitectures is shown in Figure 12.

### Bacterial culture preparation

Antimicrobial tests were conducted with the bacterial species *E. coli*. and *B. cereus.* The bacteria were cultured in sterile nutrient broth at a constant temperature of 37 °C and constant agitation in a humidified incubator for 8 h. The sample was centrifuged for 5 min at 600 rpm. Finally, the cells were resuspended in PBS buffer and slowly diluted to a concentration of ≈10^8^ CFU/mL [42]. For optical density measurements, *E. coli* (ATCC strain) and *B. cereus* (ATCC 11778) were inoculated in nutrient broth and grown overnight at 37 °C. The overnight culture was adjusted to the logarithmic growth phase, achieving an optical density of 0.20 at 600 nm, corresponding to approximately 1 × 10^8^ CFU/mL. A parallel set of samples was co-incubated with NBT (1 mg·mL^−1^) together with the nanoarchitecture under otherwise identical conditions to selectively scavenge superoxide radicals generated during nanomaterial–bacteria interaction. Following incubation, cell viability was determined via OD_600_ measurement using a UV–vis spectrophotometer. All experiments were performed in triplicate (*n* = 3), and results are expressed as mean ± standard deviation (SD).

### Antibacterial performance evaluation

**Figure 13:**
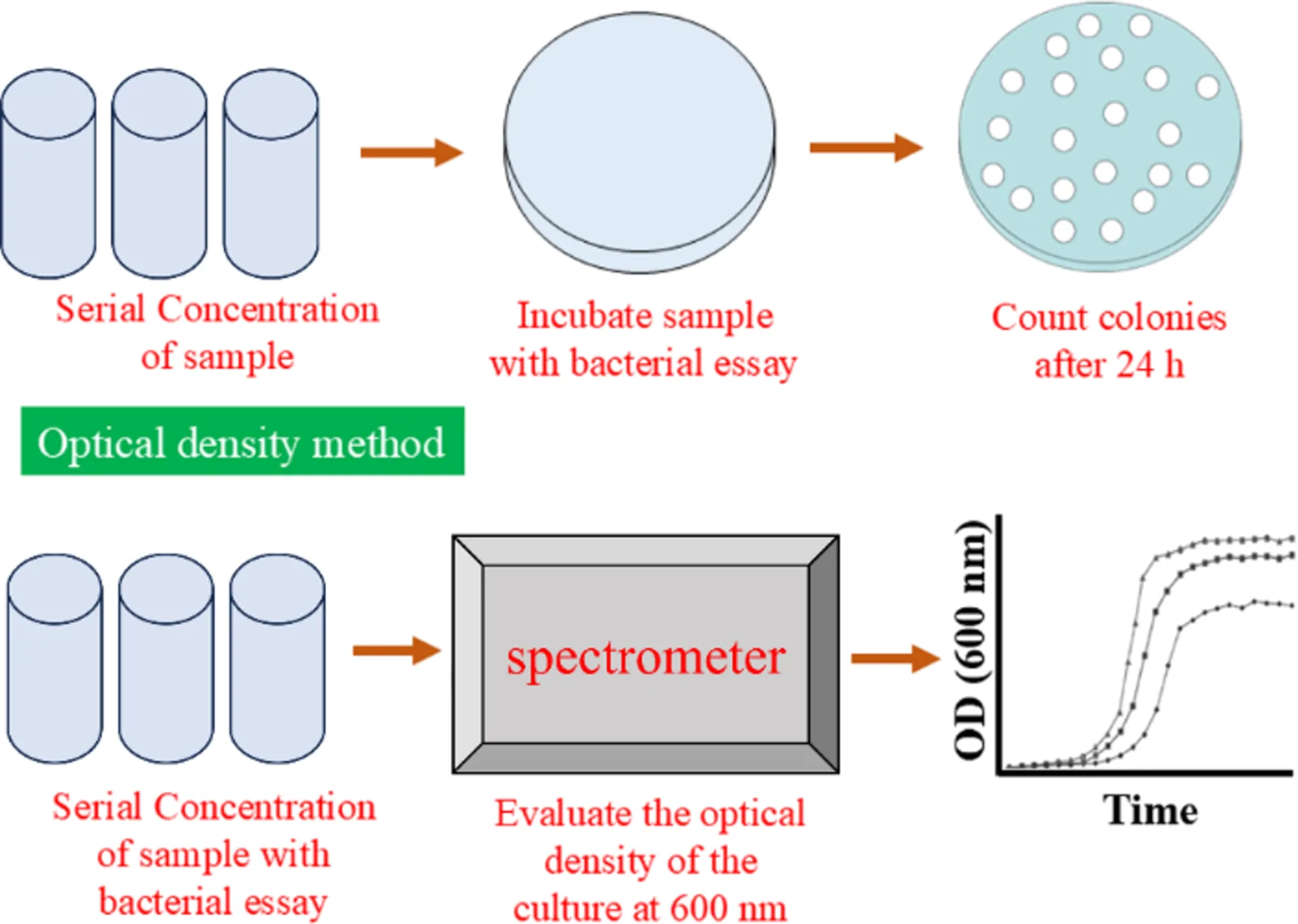
Schematic illustration of the antibacterial activity evaluation methods.

The antibacterial activity of samples against *E. coli* and *B. cereus* was systematically assessed using two complementary microbiological assays, namely, the colony forming unit (CFU) plate count method and optical density (OD_600_) growth analysis, as illustrated in Figure 13. Compared with conventional qualitative assays such as agar disk-diffusion, the OD method provides a quantitative and time-dependent evaluation of bacterial proliferation [43].

The antibacterial activity of d-MXene, graphene nanosheets, and graphene-decorated d-MXene nanoarchitectures was assessed against bacteria using a standard broth dilution assay. The bacterial culture was standardized to OD_600_ = 0.22 (≈10^8^ CFU/mL). Each nanomaterial was prepared at five concentrations (100, 200, 300, 400, and 500 µg/mL) in sterile nutrient broth. For the assay, 10 mL of sterile nutrient broth was dispersed into 50 mL flasks. Nanomaterials were added to achieve the desired final concentrations (100, 200, 300, 400, or 500 µg/mL). The standardized bacterial suspension was inoculated at 0.2% v/v (20 µL per 10 mL broth), resulting in a final bacterial concentration of approximately 2 × 10^5^ CFU/mL. The control sample contained 10 mL broth with 20 µL bacterial suspension (no nanomaterials). The incubation process of glassware was completed after 24 h at 37 °C. The growth of bacteria was observed by measuring absorbance at 600 nm after 2, 4, and 24 h. At each time point, 1 mL of sample were aseptically withdrawn from the flasks. To correct for nanomaterial absorbance, OD_600_ values of particle controls at matching concentrations and time points were subtracted from test sample values. Flasks were returned to the incubator after sampling, with total volume reduction (4 mL over four time points) considered minimal in 10 mL. To quantify viable bacteria, 100 µL samples were collected after 0, 2, 4, and finally 24 h, serially diluted in sterile nutrient broth, and plated on nutrient agar using the plate disk method. The incubation process was done at 37 °C for 24 h, and colonies were counted to determine CFU/mL. All experiments were conducted in triplicate to ensure reproducibility. Mean OD_600_ and CFU/mL values ± standard deviation were calculated [44].

### Determination of bacterial colony-forming units (CFU)

The CFU values were determined using the standard formula (Equation 5) provided below:


[5] $$\frac{\text{CFU}}{\text{mL}}=\frac{\text{number of colonies on cell culture dish×dilution factor}}{\text{plated volume}}$$

### Determination of cell viability

Cell viability represents the percentage value of living cells under defined experimental conditions, serving as a fundamental metric in assessing antibacterial performance of functional nanomaterials. In antimicrobial observation, cell viability denotes the fraction of living bacterial cells following exposure to an external agent relative to untreated controls. A measurable decline in viability is indicative of effective bacterial growth suppression or cell damage caused by the nanomaterial under test [45]. In this study, cell viability was systematically employed to probe the antibacterial efficacy of graphene nanosheets, d-MXene, and graphene-decorated d-MXene nanoarchitecture after the 24 h of inoculation against bacteria. The viability of bacterial cells post‑treatment with these nanomaterials was quantified via an optical density-based assay, with percentage viability calculated according to Equation 6. This approach enabled a rigorous evaluation of the comparative antibacterial potential of the individual components and the engineered nanoarchitecture.


[6] $$\%\text{ Cell viability}=\frac{{\text{OD}}_{\text{treatment}}}{{\text{OD}}_{\text{control}}}\times 100$$

### Statistical analysis

All antibacterial experiments were performed in triplicate (*n* = 3), and the results are expressed as mean ± standard deviation (SD). Statistical analysis was carried out using two-way analysis of variance (ANOVA) followed by Tukey’s post hoc multiple comparison test. Two-way ANOVA was employed to evaluate the simultaneous influence of material type and nanomaterial concentration on antibacterial activity. For CFU and cell viability analyses, the two independent variables were treatment material (graphene nanosheets, d-MXene, and graphene-decorated d-MXene nanoarchitecture) and nanomaterial concentration. Similarly, OD_600_ growth inhibition analysis was statistically evaluated considering material type and concentration as independent factors. Differences were considered statistically significant at *p* < 0.05.

### Characterization

PXRD patterns were recorded using an EMPYREAN diffractometer equipped with Cu Kα radiation (λ = 1.5406 Å, 40 kV) in the 2θ range of 5–80°. Raman spectra were collected using a Raman-SNOM-AFM microscope system (alpha 300-RAS, WITec GmbH, Germany) with a 785 nm excitation laser in the spectral range of 100–3000 cm^−1^. Surface morphology and elemental composition were investigated using a field-emission scanning electron microscope (Nova Nano SEM 450, FEI Company, USA) coupled with an EDS system operated at 15 kV. A TECNAI 20G2 HRTEM (FEI) operating at 200 kV was employed for transmission electron microscopy. XPS measurements were performed using a PHI 5000 Versa Probe III spectrometer equipped with a monochromatic Al Kα X-ray source (1486.6 eV) to investigate the surface chemical composition and electronic states of the synthesized nanoarchitectures. Dynamic light scattering analyzer (DLS; Malvern Zetasizer Nano ZS90, Malvern, UK) was used to analyse the diameter distribution and Zeta potential. The measurements were carried out using ethanol as the dispersant medium at 25 °C.

## Supporting Information

File 1 Additional experimental data.

## Data Availability

Data generated and analyzed during this study is available from the corresponding author upon reasonable request.

## References

[1] Thakur A, Ganesan R, Ray Dutta J (2026). Total Environ Microbiol.

[2] Wang J-W, Xun J-J, Zhao F-F (2026). Int J Nanomed.

[3] Sarac B, Yücer S, Ciftci F (2025). Nanoscale.

[4] Singh A, Kumar A, Chaurasiya N, Rani A, Gupta M, Yadav B C, Singh M P (2025). Front Nanotechnol.

[5] Murugaiyan J, Kumar P A, Rao G S, Iskandar K, Hawser S, Hays J P, Mohsen Y, Adukkadukkam S, Awuah W A, Jose R A M (2022). Antibiotics (Basel, Switz).

[6] Nusrat S, Aliyu M, Zohora F T (2025). AIMS Microbiol.

[7] Pilevar M, Dadashi Firouzjaei M, Thakur A, Nithin Chandran B S, Wahib S, Williams D, Jafarian H, Bryant C, Bedford A, Riveros A (2026). Nanoscale Horiz.

[8] Singh A, Kumar A, Singh S S, Chaurasiya N, Singh M P (2025). Mater Proc.

[9] Gnanasekar S, He X, Nagay B E, Xu K, Rao X, Duan S, Murugesan S, Barão V A R, Kang E-T, Xu L (2025). Bioact Mater.

[10] Xu Z, Meng R, Wang Y, Sun Y, Qiao J, Yao Y, Peng Q (2025). Bioengineering.

[11] Malhotra B, Keshwani A, Kharkwal H (2015). Front Microbiol.

[12] Xie M (2024). J Food Meas Charact.

[13] Singh M P, Dhumal N R, Kim H J, Kiefer J, Anderson J A (2017). J Phys Chem C.

[14] Wang M-K, Wang J, Wang F-X, Hu X-L, He X-P, James T D, Liu H, Su J-C (2026). Coord Chem Rev.

[15] Roy A S, Babu N, Hussain A (2026). Nanotechnol, Sci Appl.

[16] Li X, Li H, Liu C, Cao Y, Xu Z, Fu F, Yu J (2026). ACS Nano.

[17] Tiwari P R, Singh R P, Bharati K, Yadav A C, Kumar K, Yadav B C, Kumar S, Singh M P (2022). Phys Status Solidi A.

[18] Huang J, Zhang D, Zhu C, Chen S, Wang Y, Han K, Ci S, Lv Y (2025). RSC Adv.

[19] Park S, Byun H J, Lee J Y (2026). Biomater Res.

[20] Adhami A, Mozafari M, Soroush M (2026). Commun Mater.

[21] Gao R, Xia Z, Li P, Bian R, Zhang N, Lu J, Fan C, Liu L, Deng Y (2026). Surf Interfaces.

[22] Singh M P, Kumar A, Singh A, Singh S S, Chaurasiya S K (2025). Mater Proc.

[23] Al-Aoh H A, Panneerselvam C, Alghamdi A M, Alasmari A, Mohammedsaleh Z M (2026). Analyst.

[24] Korniienko V, Gogotsi O, Varava Y, Zandersone B, Deineka V, Husak Y, Diedkova K, Solodovnyk O, Kukurika V, Dukhnovskiy S (2026). ACS Appl Nano Mater.

[25] Gupta A K, Verma Y L, Singh M P, Singh R K (2016). J Solid State Chem.

[26] Sarkar S, Hossain S A, Chakraborty P, Ghosh S, Bhattacharya S, Mukherjee S, das P (2024). Surf Interfaces.

[27] Rasool K, Helal M, Ali A, Ren C E, Gogotsi Y, Mahmoud K A (2016). ACS Nano.

[28] Kumar A, Singh A, Singh M P (2025). Mater Proc.

[29] Kumar A, Singh A, Chaurasiya N, Singh P, Singh M P (2026). Mater Technol (Abingdon, U K).

[30] Fatimah S, Ragadhita R, Al Husaeni D F, Nandiyanto A B D (2022). ASEAN J Sci Eng.

[31] Sutapa I W, Wahid Wahab A, Taba P, Nafie N L (2018). J Phys: Conf Ser.

[32] Tian H, Ma Y, Li Z, Cheng M, Ning S, Han E, Xu M, Zhang P-F, Zhao K, Li R (2023). Nature.

[33] Iqbal A, Hamdan N M (2021). Materials.

[34] Gopal T S, Alzahrani K E, Assaifan A K, Albrithen H, Alodhayb A, Muthuramamoorthy M, Pandiaraj S, Grace A N (2022). Sci Rep.

[35] Singh A, Kumar A, Rani A, Shankar U, Chaurasiya N, Yadav B C, Singh M P (2026). J Mater Sci: Mater Electron.

[36] Santhosh C, Kollu P, Doshi S, Sharma M, Bahadur D, Vanchinathan M T, Saravanan P, Kim B-S, Grace A N (2014). RSC Adv.

[37] Chaudhary R, Singh N B, Nagpal G, Saah F K (2024). The Microbe.

[38] Chandel M, Jakubczak M, Purbayanto M A K, Górnik A, Basior W, Moszczyńska D, Tabassum A, Naguib M, Jastrzębska A M (2025). Nanoscale.

[39] Akinay Y, Karatas E, Ruzgar D, Akbari A, Baskin D, Cetin T, Kazici H C, Topuz M (2025). Sci Rep.

[40] Krzepiłko A, Matyszczuk K M, Święciło A (2023). Pathogens.

[41] García M, Martínez-Flórez I, Solano-Gallego L, García N, Ordeix L (2024). Vet Sci.

[42] Zou F, Zhou H, Jeong D Y, Kwon J, Eom S U, Park T J, Hong S W, Lee J (2017). ACS Appl Mater Interfaces.

[43] Balouiri M, Sadiki M, Ibnsouda S K (2016). J Pharm Anal.

[44] Salmi M S, Ahmed U, Aslfattahi N, Rahman S, Hardy J G, Anwar A (2022). RSC Adv.

[45] Sorourian G, Pourmadadi M, Yazdian F, Rashedi H, Nigjeh M N, Sorourian M, Fathi-karkan S, Rahdar A, Pandey S (2025). Bionanosci.

