# Supplementary material for: Bottom-up assembly of graphene-decorated d-MXene nanoarchitecture for enhanced antibacterial activity against Escherichia coli and Bacillus cereus
Source: Beilstein J Nanotechnol. 2026 Oct 22;17:1453–77. doi: 10.3762/bjnano.17.100 (PMC13698924; doi:10.3762/bjnano.17.100)
Supplement: Supplementary file 1 [file Beilstein_J_Nanotechnol-17-1453-s001.pdf]

BEILSTEIN JOURNAL OF NANOTECHNOLOGY

Supporting Information

for

Bottom-up assembly of graphene-decorated d-MXene nanoarchitecture for enhanced antibacterial activity against *Escherichia coli* and *Bacillus cereus*

Avdhesh Kumar, Ankit Singh, Ishani Bharti, Chandra Bhushan, Ram Naraian and Manish Pratap Singh

Beilstein J. Nanotechnol. **2026,** *17,* 1453–1477. doi:10.3762/bjnano.17.100

Additional experimental data

License and Terms: This is a supporting information file under the terms of the Creative Commons Attribution License (https://creativecommons.org/licenses/by/4.0). Please note that the reuse, redistribution and reproduction in particular requires that the author(s) and source are credited and that individual graphics may be subject to special legal provisions.
The license is subject to the *Beilstein Journal of Nanotechnology* terms and conditions: (https://www.beilstein-journals.org/bjnano/terms)

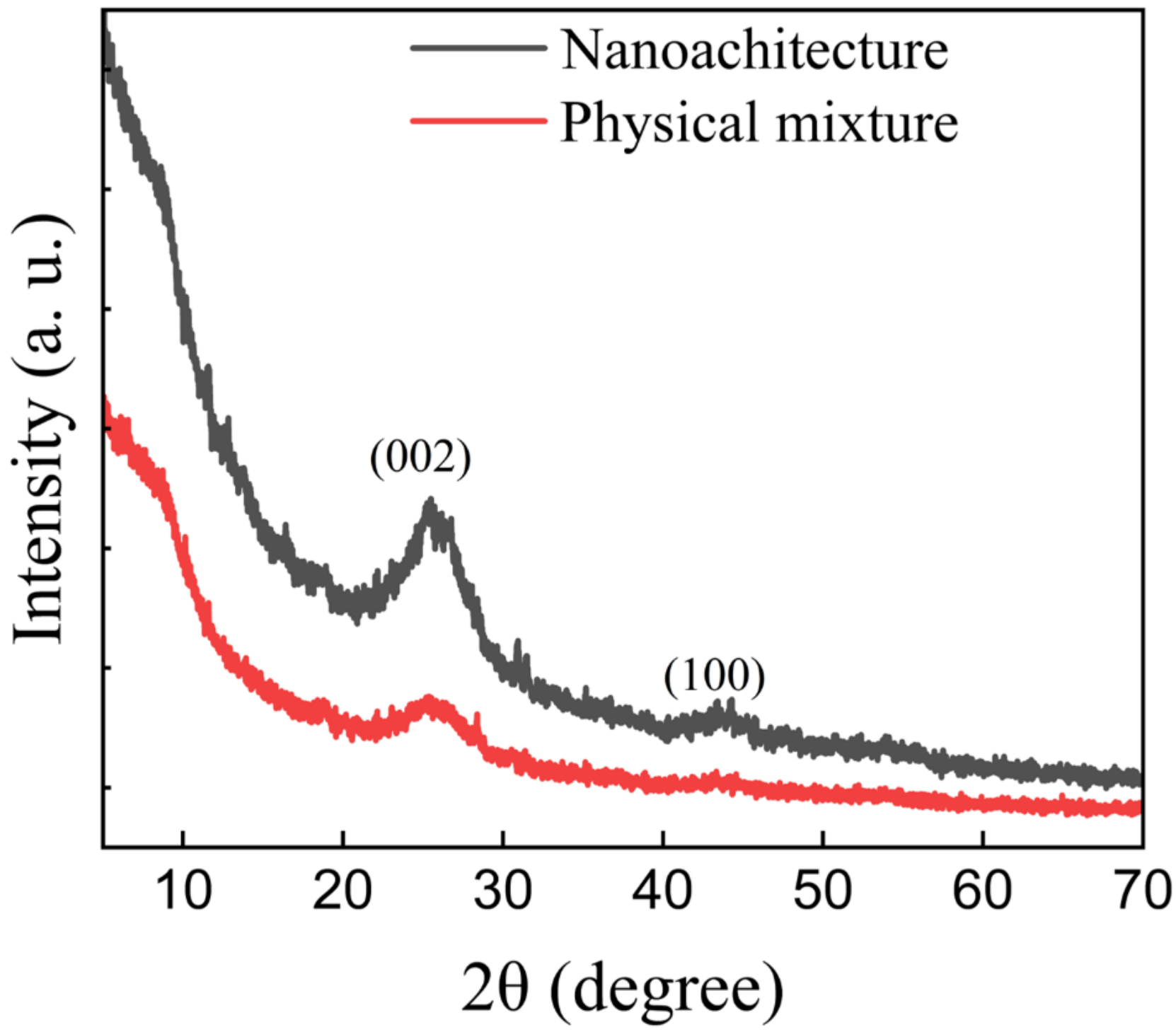

Figure S1: XRD comparison of nanoarchitecture with physical mixture.

Comparative PXRD measurements were performed on the ultrasonication-assisted nanoarchitecture and a physical mixture prepared using the same graphene and d-MXene composition (Figure S1), in order to further verify that the graphene-decorated d-MXene material is not merely a simple physical mixture. Both samples exhibit a broad reflection centred at 26.2°, corresponding to the characteristic graphitic (002) plane of graphene, consistent with the intentionally graphene-rich composition of the nanoarchitecture. As the characteristic d-MXene (002) reflection is typically reported near 6-9° 2θ, it is not clearly resolved within the presently scanned 2θ range and is obscured by the dominant graphene signal. Distinct differences between the nanoarchitecture and the physical mixture are nonetheless observed in the 20-30° region, where the nanoarchitecture displays a sharper and more resolved graphitic (002) reflection relative to the physical mixture. This sharpening suggests improved graphitic stacking order arising from the interfacial assembly process, rather than reflecting a separate d-MXene contribution. A weak additional peak observed at ~43° in the nanoarchitecture, assigned to the (100) plane of graphitic carbon, is attributed to minor oxidation occurring during the sonication process. In contrast, the physical mixture retains a comparatively broad and less resolved (002) feature, consistent with the individual components retaining their native, loosely associated stacking without genuine interfacial coupling.

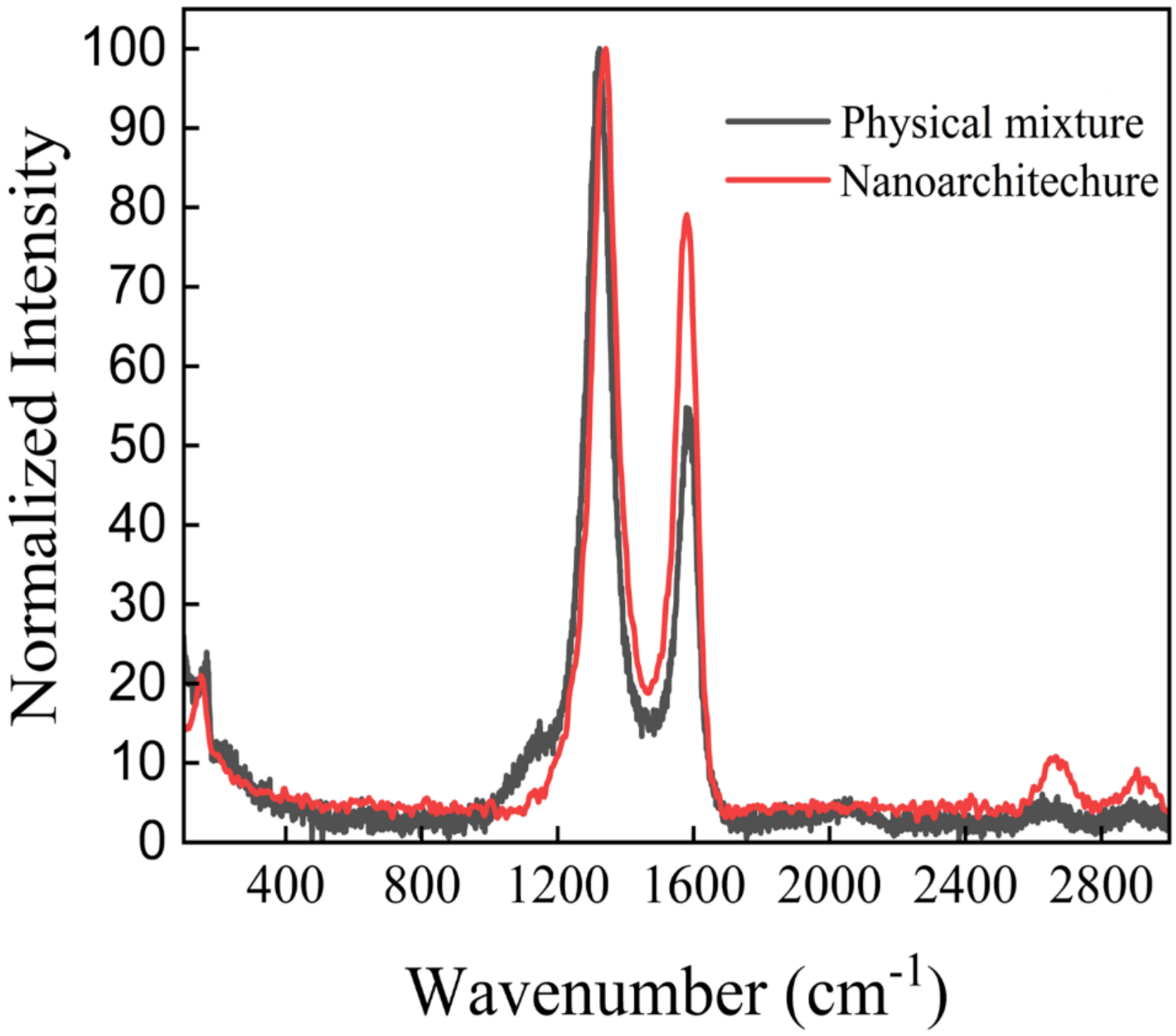

Figure S2: Raman spectra of nanoarchitecture and physical mixture.

Complementary Raman spectroscopy (Figure S2) was performed to further probe interfacial interaction between graphene and d-MXene. The nanoarchitecture exhibits a clearly enhanced band in the 1500-1700 cm^{-1} region relative to the physical mixture, along with subtle differences in the low-wavenumber and 2500-2900 cm^{-1} regions. More importantly, the I_D/I_G ratio decreases from 1.83 for the physical mixture to 1.27 for the graphene-decorated d-MXene nanoarchitecture. The lower I_D/I_G ratio indicates that the ultrasonication-assisted assembly does not introduce additional structural disorder into the graphene framework. Instead, it suggests a more ordered graphitic structure arising from uniform interfacial organization and intimate contact between graphene and d-MXene nanosheets. The decrease in the I_D/I_G ratio is consistent with suppression of random sheet aggregation and the formation of a structurally integrated nanoarchitecture rather than a simple mechanical mixture. Taken together with the PXRD results, these spectral differences are attributed to genuine interfacial coupling between graphene and d-MXene arising from the ultrasonication-assisted assembly process, rather than to compositional variation alone.

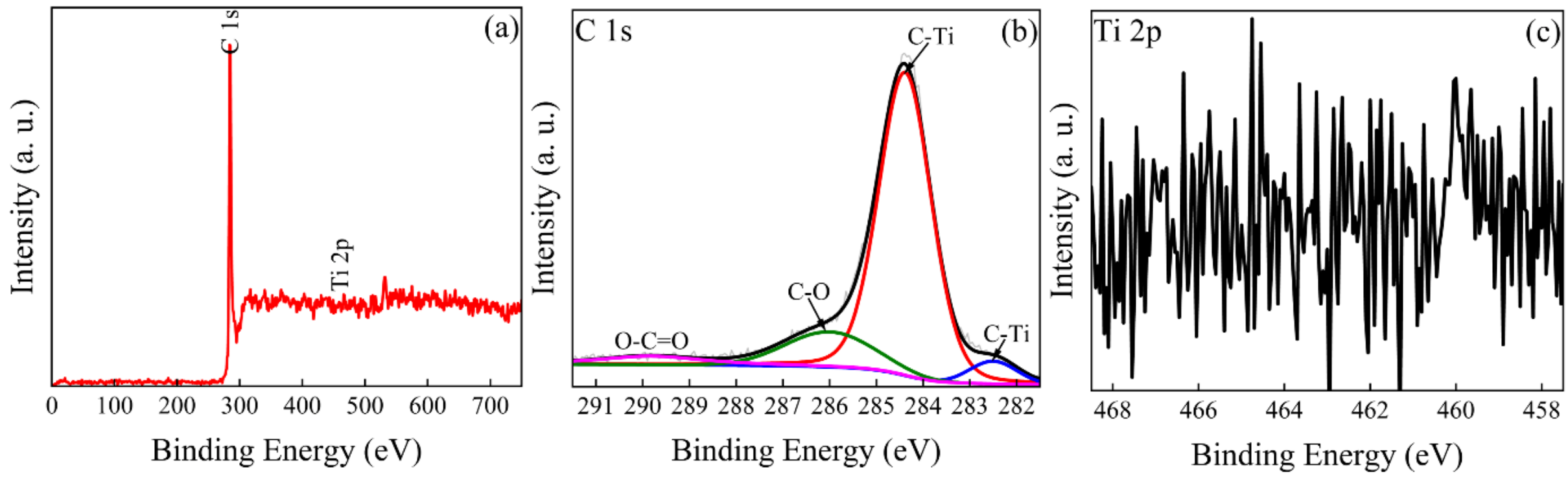

Figure S3: XPS analysis of the physical mixture of graphene and d-MXene: (a) survey spectrum, (b) high-resolution C 1s spectrum, (c) high-resolution Ti 2p spectrum.

Further supporting evidence is provided by high-resolution XPS comparison between the nanoarchitecture (Figure 5) and the physical mixture control (Figure S3). While the C 1s C-Ti component appears at a comparable binding energy in both samples (282.4 eV for the physical mixture vs. 282.9 eV for the nanoarchitecture), confirming retention of the native d-MXene carbide framework, a clear difference is observed in the Ti 2p and O 1s regions: the physical mixture shows a poorly resolved, high-noise Ti 2p signal with no clearly identifiable surface-termination components in O 1s, whereas the nanoarchitecture exhibits a well-resolved Ti $2p_{3/2}$/ Ti $2p_{1/2}$ doubles and three distinct O 1s components (Ti-O, C-Ti-$(OH)_x$, O=C-OH) characteristic of d-MXene surface terminations. Given the comparable graphene: d-MXene ratio in both samples, this indicates improved surface accessibility of the d-MXene component following ultrasonication-assisted assembly, consistent with the PXRD and Raman evidence for suppressed nanosheet restacking, rather than reflecting a difference in composition ratio.
